# New insights into the occurrence of continuous cropping obstacles in pea (*Pisum sativum* L.) from soil bacterial communities, root metabolism and gene transcription

**DOI:** 10.1186/s12870-023-04225-8

**Published:** 2023-04-28

**Authors:** Lei Ma, Shaoying Ma, Guiping Chen, Xu Lu, Ruonan Wei, Ling Xu, Xiaojie Feng, Xiaoming Yang, Qiang Chai, Xucheng Zhang, Sheng Li

**Affiliations:** 1 State Key Laboratory of Arid land Crop Science, College of Agronomy, Gansu Agricultural University, Lanzhou, China; 2 Basic Experimental Teaching Center, Gansu Agricultural University, Lanzhou, 730070 China; 3 College of Horticulture, Gansu Agricultural University, Lanzhou, 730070 China; 4 College of Life Science and Technology, Gansu Agricultural University, Lanzhou, 730070 China; 5 Crop Research Institute, Gansu Academy of Agricultural Sciences, Lanzhou, 730070 China; 6 Dryland Agricultural Institute, Gansu Academy of Agricultural Sciences, Lanzhou, 730070 China; 7 State Key Laboratory of Arid land Crop Science, College of Life Science and Technology, Gansu Agricultural University, Lanzhou, 730070 China

**Keywords:** Pea, Continuous cropping obstacles, Bacterial community structure and diversity, Metabolome, Transcriptome, Correlation

## Abstract

**Background:**

Continuous cropping is a significant obstacle to sustainable development in the pea (*Pisum sativum* L.) industry, but the underlying mechanisms of this remain unclear. In this study, we used 16 S rDNA sequencing, transcriptomics, and metabolomics to analyze the response mechanism of roots and soil bacteria to continuous cropping and the relationship between soil bacteria and root phenotypes of different pea genotypes (Ding wan 10 and Yun wan 8).

**Results:**

Continuous cropping inhibited pea growth, with a greater effect on Ding wan 10 than Yun wan 8. Metabolomics showed that the number of differentially accumulated metabolites (DAMs) in pea roots increased with the number of continuous cropping, and more metabolic pathways were involved. Transcriptomics revealed that the number of differentially expressed genes (DEGs) increased with the number of continuous cropping. Continuous cropping altered the expression of genes involved in plant-pathogen interaction, MAPK signal transduction, and lignin synthesis pathways in pea roots, with more DEGs in Ding wan 10 than in Yun wan 8. The up-regulated expression of genes in the ethylene signal transduction pathway was evident in Ding wan 10. Soil bacterial diversity did not change, but the relative abundance of bacteria significantly responded to continuous cropping. Integrative analysis showed that the bacteria with significant relative abundance in the soil were strongly associated with the antioxidant synthesis and linoleic acid metabolism pathway of pea roots under continuous cropping once. Under continuous cropping twice, the bacteria with significant relative abundance changes were strongly associated with cysteine and methionine metabolism, fatty acid metabolism, phenylpropanoid biosynthesis, terpenoid backbone biosynthesis, linoleic acid, and amino sugar and nucleotide sugar metabolism.

**Conclusion:**

Ding wan 10 was more sensitive to continuous cropping than Yun wan 8. Continuous cropping times and pea genotypes determined the differences in root metabolic pathways. There were common metabolic pathways in the two pea genotypes in response to continuous cropping, and the DEGs and DAMs in these metabolic pathways were strongly associated with the bacteria with significant changes in relative abundance in the soil. This study provides new insights into obstacles to continuous cropping in peas.

**Supplementary Information:**

The online version contains supplementary material available at 10.1186/s12870-023-04225-8.

## Background

Limited arable land area and increased market demand have led to the widespread practice of continuous cropping in modern agriculture, which can cause obstacles to continuous cropping resulting in crop growth inhibition and yield reduction. This can cause significant economic losses and seriously impede the sustainable development of modern agriculture [1–4]. Therefore, researching continuous cropping obstacles is of great practical importance. Crop growth, development, and immunity are closely related to soil microorganisms [5, 6], and the obstacles to continuous cropping result from the integrated plant-soil-microorganism interaction [7]. It is known that changes in soil microbial community structure and diversity are key factors in the occurrence of continuous cropping obstacles [8, 9]. Although many studies have been reported, the results differ due to the influence of factors such as soil types, continuous cropping times, crop type, and genotype [10]. Therefore, it is crucial to further study changes in soil microbial communities under specific conditions to better understand the obstacles to continuous cropping.

Bacteria constitute the largest component of the soil microbial community and play a crucial role in soil structure and biological interactions [11, 12]. The present study found that bacterial community composition was susceptible to crop root secretions [13, 14], and changes in the rhizosphere bacterial community inevitably affected crop growth [15]. Previous studies investigating continuous cropping of peanuts, found that it reduced soil bacterial diversity, down-regulation of auxin and cytokinin synthesis genes in peanut roots, and up-regulation of genes related to abscisic acid, jasmonic acid, salicylic acid, and the ethylene signal transduction pathway [16]. However, it remains to be confirmed whether differential expression of these genes leads to changes in metabolite abundance, thus affecting phenotypes. Moreover, the growth and development of crops under continuous cropping conditions are influenced by multiple metabolic pathways and a variety of complex metabolites, and it is worth investigating whether these pathways and metabolites respond to changes in soil bacteria.

The pea crop (*Pisum sativum* L.) is the fourth largest legume crop in the world and a strategic commodity for global food security [17], but there are continuous cropping obstacles in cultivation. The pea plants yield and soil microbial biomass are known to decrease during continuous cropping compared with rotation [18]. However, it has not yet been reported whether pea plants respond to changes in the soil bacterial communities and thus cause changes of plant phenotypes and whether this is affected by the pea genotype.

Root systems are in direct contact with the soil, and are the first organs to perceive changes in the soil environment that can subsequently affect plant growth. Therefore, it is crucial to study the response of pea root systems to continuous cropping. We hypothesized that changes in the soil bacterial community under continuous cropping might cause changes in the expression of some metabolic pathways and related genes in pea roots, leading to obstacles to continuous cropping and that the degree of harm varied according to different pea genotypes and continuous cropping times. In this study, we selected two pea genotypes and used soil bacterial 16 S rDNA, pea root transcriptomics, and metabolomics to investigate the effects of continuous cropping on the soil bacterial community and its relationships with changes in pea plant root phenotype. The findings of this study provide a new theoretical basis for elucidating the mechanism of obstacles to continuous cropping in pea plants.

## Results

### Continuous cropping soils inhibited the growth of pea plants

**Fig. 1:**
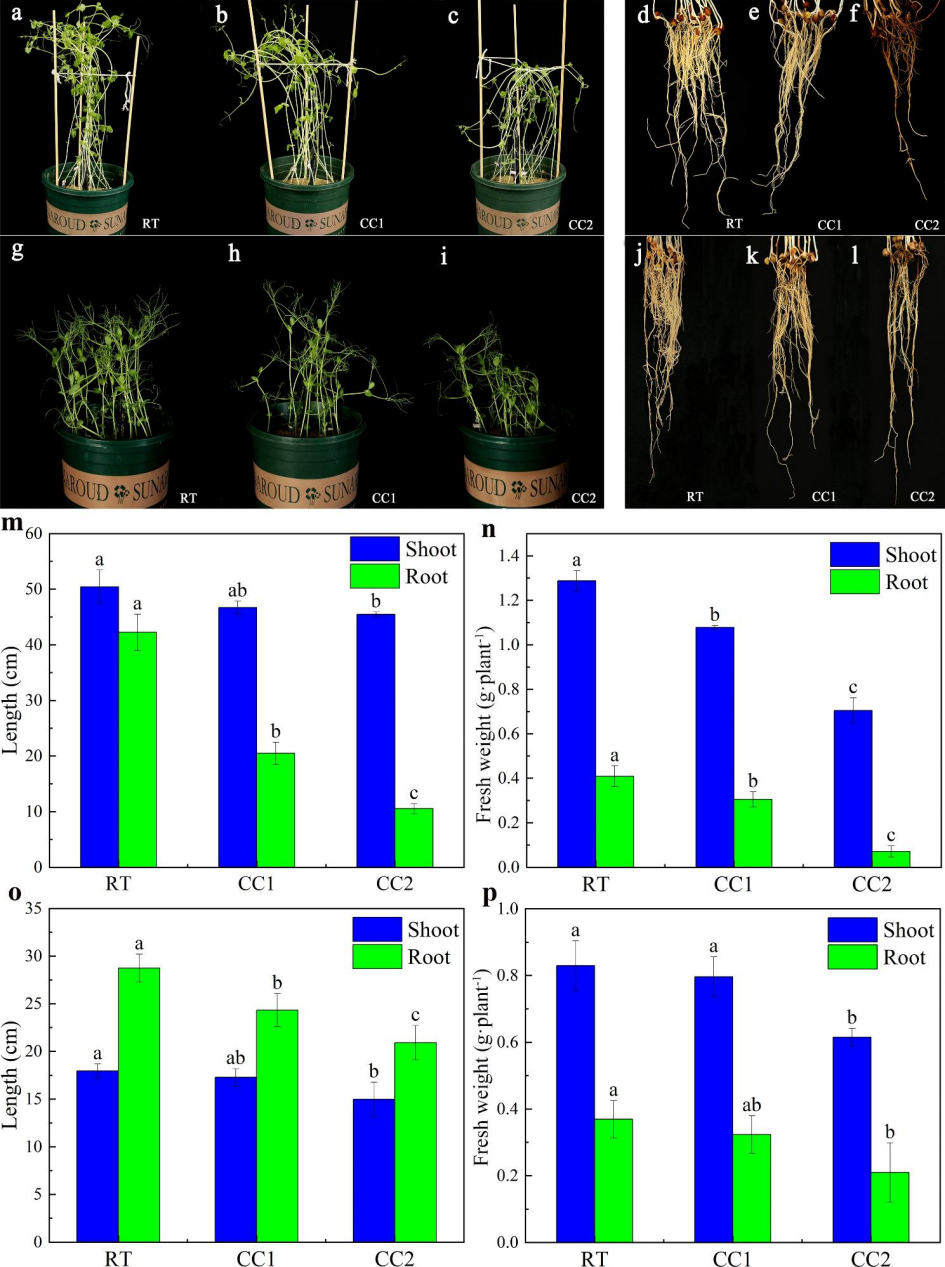
Phenotypic variation characteristics of pea plants under different continuous cropping conditions. **a-c**: Shoot phenotype of Ding wan 10. **d-f**: Root phenotype of Ding wan 10. **g-i**: Shoot phenotype of Yun wan 8. **j-l**: Root phenotype of Yun wan 8. **m**: Shoot height and root length of Ding wan 10. **n**: Shoot (or root) fresh weight of Ding wan 10. **o**: Shoot height and root length of Yun wan 8. **p**: Shoot (or root) fresh weight of Yun wan 8. **RT**: Rotation treatment. **CC1**: Continuous cropping once treatment. **CC2**: Continuous cropping twice treatment. Different letters indicate that the means differ significantly (*P* < 0.05) according to Duncan’s multiple range test

Pot experiments showed that the growth of peas under continuous cropping was inhibited (Fig. 1a-c, g-i). Morphological observations and index analysis of the material collected from the second pot experiment revealed that the continuous cropping treatments reduced shoot height, shoot (or root) fresh weight, and root length of Ding wan 10, and the degree of inhibition increased under continuous cropping twice treatment (CC2) (Fig. 1m, n). Compared with the rotation treatment (RT), continuous cropping once treatment (CC1) did not significantly affect the growth of Yun wan 8 (except root length), but all indexes were significantly decreased under CC2 (Fig. 1o, p). This indicated that Ding wan 10 was more sensitive to continuous cropping treatments, with growth being inhibited under CC1 and the degree of inhibition being aggravated under CC2.

Yun wan 8 was more tolerant of continuous cropping and its growth was not significantly inhibited under CC1, but was significantly inhibited by CC2. Phenotypic analysis showed that the root system of pea plants changed (Fig. 1d-f, j-l), especially after CC2, the root system of the two pea genotypes aged early, and the degree of aging was increased in Ding wan 10 (Fig. 1d-f).

### Continuous cropping led to changes in the metabolic level of pea roots

**Fig. 2:**
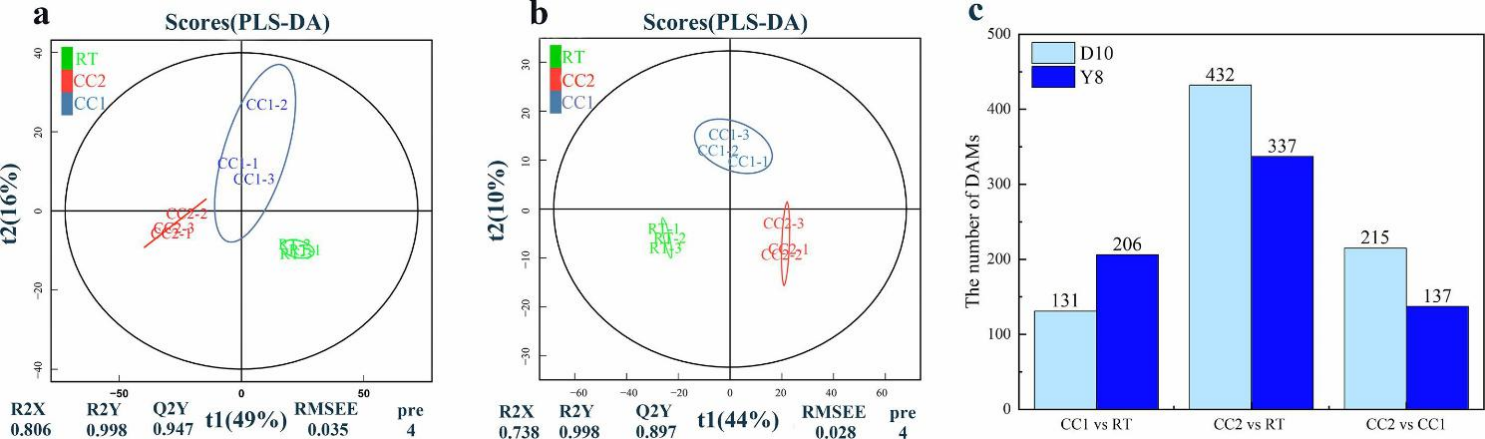
Analysis of root metabolites differences of pea plants under different continuous cropping conditions. **a** and **b** are the partial least squares discriminant analysis (PLS-DA) of Ding wan 10 and Yun wan 8, respectively. **c**: Number of DAMs. **RT**: Rotation treatment. **CC1**: Continuous cropping once treatment. **CC2**: Continuous cropping twice treatment

Plant phenotypes undergo changes that are inevitably influenced by their metabolite levels. To better comprehend how continuous cropping affects the root metabolism of two pea genotypes, we performed a metabolomic analysis using LC-MS/MS. The score plots, based on the PLS-DA model, demonstrated spatial differences between the data of the different treatments, indicating differences between the groups (Fig. 2a, b). We applied thresholds (VIP > 1, |FC| > 1, *P* < 0.05) to screen for DAMs. In the Ding wan 10, we found 131, 432, and 215 DAMs between CC1 and RT, CC2 and RT, and CC2 and CC1, respectively (Fig. 2c). In the Yun wan 8, we found 206, 337, and 137 DAMs between CC1 and RT, CC2 and RT, and CC2 and CC1, respectively (Fig. 2c). Under CC2, we observed that the number of DAMs for Ding wan 10 was higher than that of Yun wan 8, indicating that continuous cropping had a greater impact on Ding wan 10 than on Yun wan 8.

Autotoxins were identified as the primary cause of continuous cropping obstacles. Our analyses revealed no change in the levels of cinnamic acid and its derivatives, ferulic acid, and coumaric acid analogs in CC1 compared to the RT of Ding wan 10. However, in CC2, the levels of these metabolites increased (Table S1). The levels of cinnamic acid derivatives, ferulic acid, and coumaric acid derivatives were higher in CC2 than in CC1 and RT of Ding wan 10 (Table S1). In Yun wan 8, there was no change in the levels of ferulic acid and coumaric acid compared with the RT (Table S2). The levels of 4-methoxy cinnamic acid and 3-[(1-carboxy vinyl) oxy] benzoic acid were higher in CC2 than in CC1 of Yun wan 8, but the difference was not significant (Table S2). These substances have been proven autotoxicity in many crops and may be potential autotoxins in pea roots. Our results indicate that continuous cropping increased levels of potential autotoxins in pea roots, which were secreted into the rhizosphere and could also affect plant growth by influencing rhizosphere microorganisms. We also found differences between the two pea genotypes, with fewer differential potential autotoxin species in Yun wan 8 than Ding wan 10.

We performed a Kyoto Encyclopedia of Genes and Genomes (KEGG) classification analysis to understand the metabolic pathways involved in DAMs. In Ding wan 10, compared with the RT, 41 identical metabolic pathways were involved in DAMs between CC1 and CC2, and 33 metabolic pathways were specific to CC2 (Fig. S1a, b). Compared with the CC1, CC2 had a total of 55 metabolic pathways, 18 of which were identical to the specific metabolic pathways between CC2 and RT (Fig. S1c). These 18 unique metabolic pathways involved plant hormone signal transduction, amino acid metabolism, biosynthesis of other secondary metabolites, carbohydrate metabolism, lipid metabolism, cofactor and vitamin metabolism, terpenoids, and polyketides metabolism (Fig. S1b, c). In Yun wan 8, compared with the RT, 49 identical metabolic pathways were involved in DAMs between CC1 and CC2, and 14 metabolic pathways were specific to CC2 (Fig. S1d, e). Compared with the CC1, CC2 had a total of 53 metabolic pathways, four of which were identical to the specific metabolic pathways between CC2 and RT (Fig. S1f). Among them, the four unique metabolic pathways involved plant hormone signal transduction, biosynthesis of other secondary metabolites, lipid metabolism, and cofactor and vitamin metabolism (Fig. S1e, f).

**Fig. 3:**
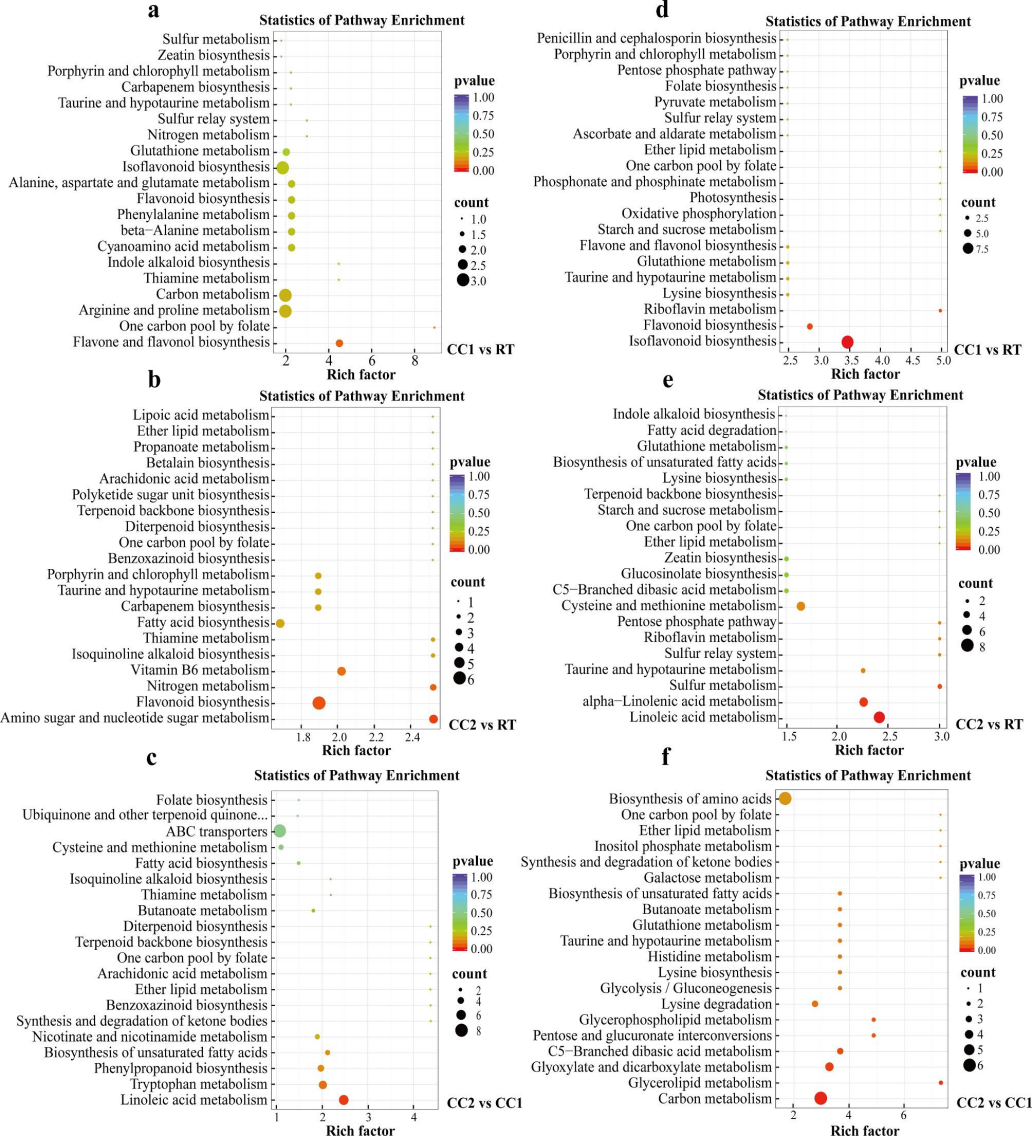
KEGG enrichment analysis of pea roots metabolomics under different continuous cropping conditions. **a**, **b** and **c** are Ding wan 10. **d**, **e and f** are Yun wan 8. **RT**: Rotation treatment. **CC1**: Continuous cropping once treatment. **CC2**: Continuous cropping twice treatment

The KEGG enrichment analysis revealed that under CC2 of Ding wan 10, flavonoid biosynthesis, nitrogen metabolism, and amino sugar and nucleotide sugar metabolism were enriched **(**Fig. 3b**)**. Under CC1, the biosynthetic pathways of flavone and flavonol were significantly enriched **(**Fig. 3a**)**. Fatty acid metabolic pathways were significantly enriched in CC2 compared to CC1 **(**Fig. 3c**)**. In Yun wan 8, isoflavonoid biosynthesis, flavonoid biosynthesis and riboflavin metabolism were enriched under CC1 **(**Fig. 3d**)**. The metabolic pathways enriched under CC2 were linoleic acid metabolism, alpha-linolenic acid metabolism, and sulfur metabolism **(**Fig. 3e**)**. Carbon metabolism and glycerolipid metabolism were significantly enriched in CC2 compared to CC1 **(**Fig. 3f**)**. These results indicate that the different durations of continuous cropping led to responses in different metabolic pathways in pea roots. Additionally, the metabolic pathways enriched only under CC2 may represent the specific response of peas to severe continuous cropping stress.

### Response characteristics of pea root transcription level to continuous cropping

**Fig. 4:**
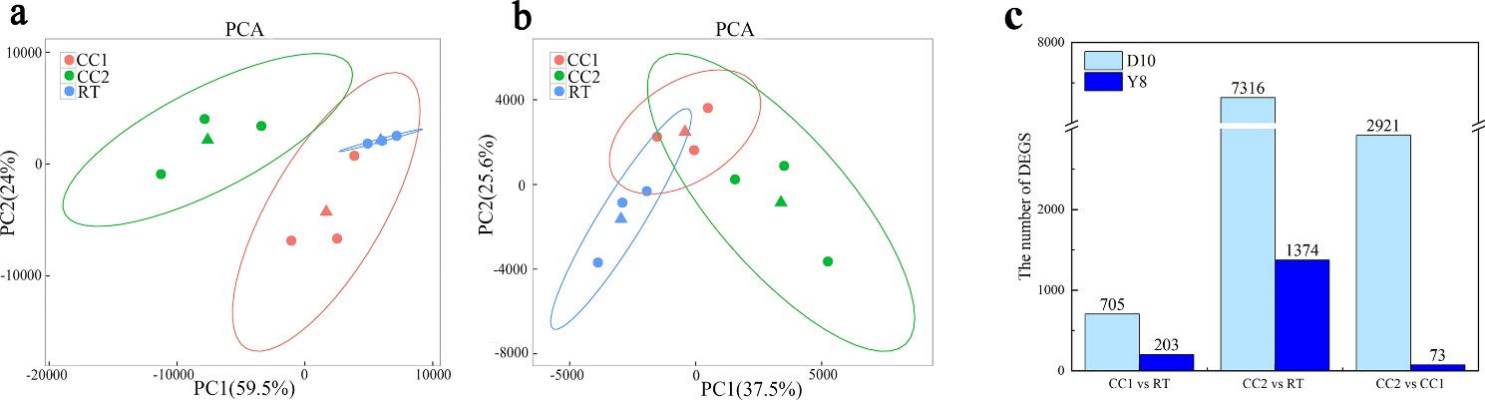
Analysis of DEGs in pea roots under different continuous cropping conditions. **a** and **b** are principal component analysis (PCA) of Ding wan 10 and Yun wan 8, respectively. **c**: Number of DEGs. **RT**: Rotation treatment. **CC1**: Continuous cropping once treatment. **CC2**: Continuous cropping twice treatment

The expression of related genes regulates metabolite changes. Therefore, we compared the transcriptomes of pea roots under different continuous cropping conditions, verified the data quality through principal component analysis (Fig. 4a, b), and performed DEGs screening (FDR < 0.05, |FC| > 1.5). In Ding wan 10, compared with the RT, there were 705 DEGs in CC1, 7316 DEGs in CC2, and 2921 DEGs in CC2 compared to CC1 (Fig. 4c). In Yun wan 8, there were 203 DEGs in CC1, 1374 DEGs in CC2, and 73 DEGs in CC2 compared to CC1 (Fig. 4c). Numerous genes were differentially expressed in the two pea genotypes under CC2, and the number of DEGs in Ding wan 10 was higher than in Yun wan 8.

**Fig. 5:**
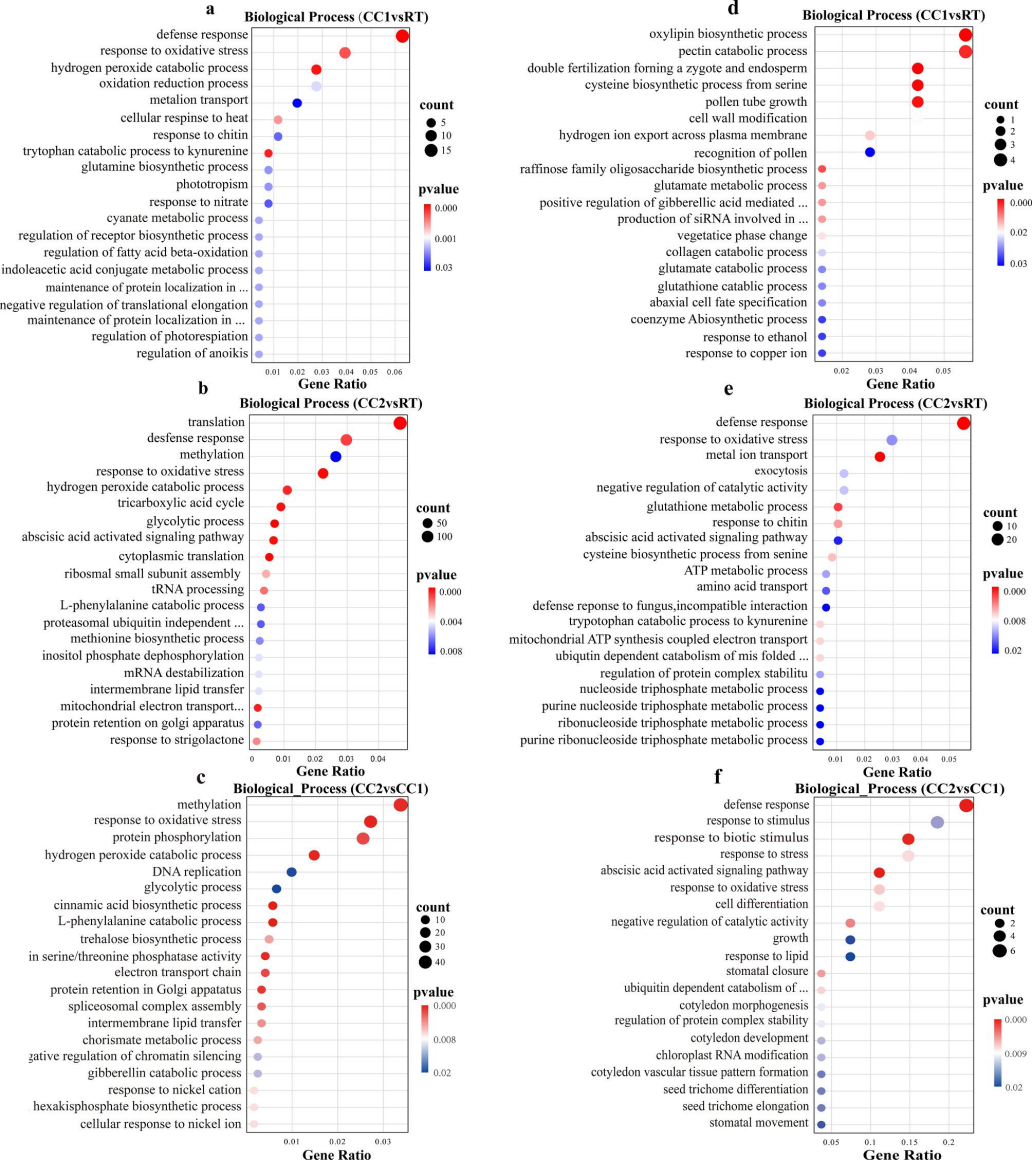
Biological process in gene ontology (GO) classification of DEGs. **a**, **b** and **c** are Ding wan 10. **d, e** and **f** are Yun wan 8. **RT**: Rotation treatment. **CC1**: Continuous cropping once treatment. **CC2**: Continuous cropping twice treatment

The gene ontology (GO) functional enrichment analysis of DEGs revealed that in the biological process, the two continuous cropping treatments in Ding wan 10 significantly induced pea roots defense and oxidative stress response processes. Compared with CC1, the CC2 treatment induced the oxidative stress response process (Fig. 5a, b, c). In Yun wan 8, the defense response was not induced under CC1, but CC2 significantly induced both the defense and oxidative stress response processes (Fig. 5d, e, f).

**Fig. 6:**
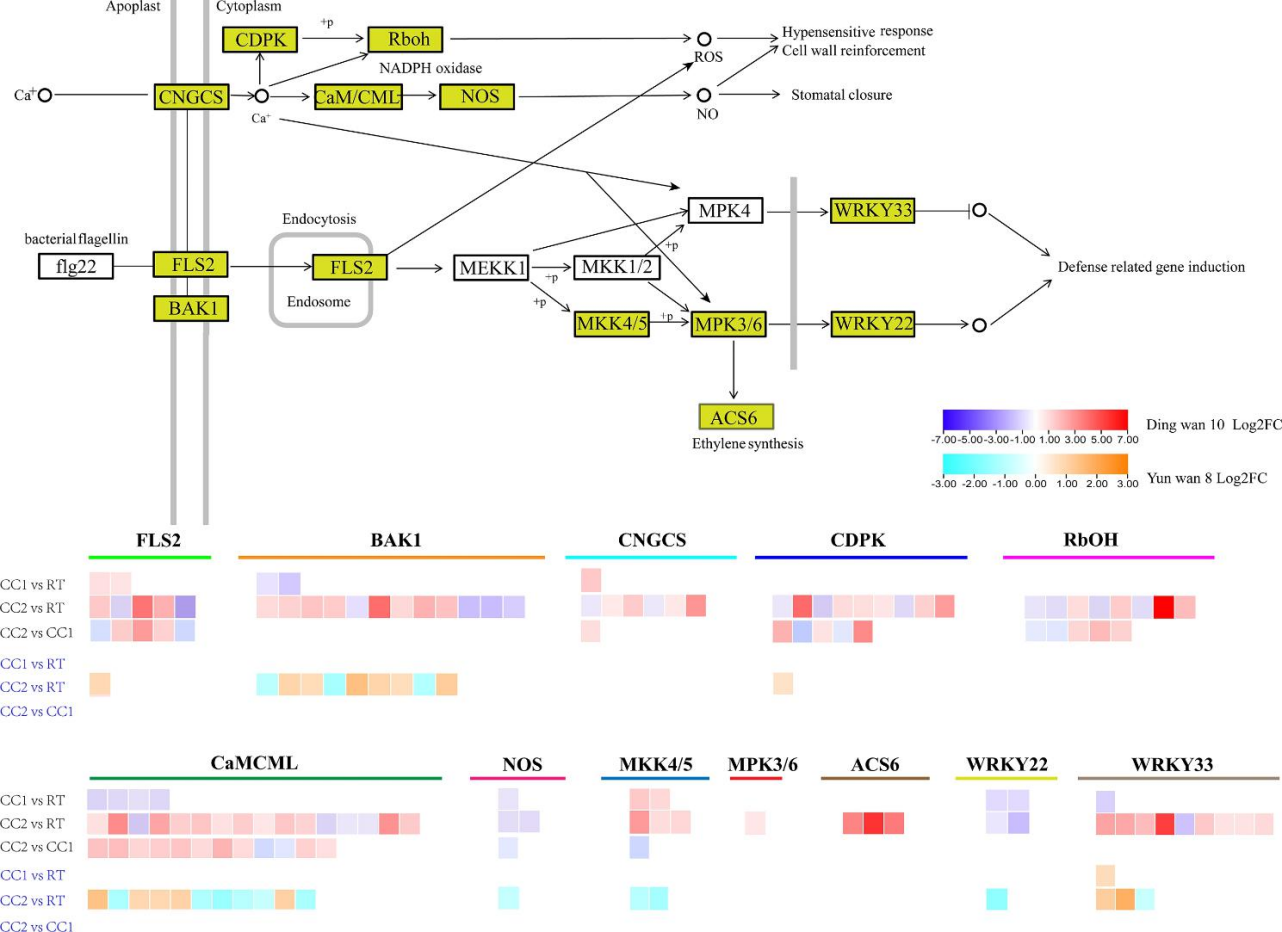
Enrichment of DEGs in plant-pathogen interactions. **RT**: Rotation treatment. **CC1**: Continuous cropping once treatment. **CC2**: Continuous cropping twice treatment

We performed a KEGG classification analysis to understand the metabolic pathways involved in the DEGs. The most DEGs between CC1 and RT, CC2 and RT, CC2 and CC1 in Ding wan 10, and CC2 and RT in Yun wan 8 were found in the plant-pathogen interaction pathway (Fig. S2a-c, e). Meanwhile, there are a large number of DEGs involved in plant-pathogen interactions between CC1 and RT, and CC2 and CC1 in Yun wan 8 (Fig. S2d, f). In this metabolic pathway, *FLS2* and *BAK1* were differentially expressed between CC2 and RT in Ding wan 10 and Yun wan 8, with more DEGs in Ding wan 10 (Fig. 6). Additionally, calcium-dependent and MAPK signaling pathways involved in this metabolic pathway were activated to varying degrees. *CNGCS* and *Rboh* were differentially expressed only in Ding wan 10, and many DEGs existed between CC2 and RT, CC2 and CC1 (Fig. 6). *CaMCML* was differentially expressed between different treatments of Ding wan 10, but only between CC2 and RT in Yun wan 8 (Fig. 6). *NOS* and *MKK4/5* were differentially expressed between CC1 and RT, CC2 and RT, CC2 and CC1 in Ding wan 10, and CC2 and RT in Yun wan 8 (Fig. 6). Moreover, the transcription factors *WRKY22* and *WRKY33* were differentially expressed in the continuous cropping treatments of both Ding wan 10 and Yun wan 8 (Fig. 6). The number of DEGs involved in the plant-pathogen interaction pathway and the calcium-dependent and MAPK signaling pathways in Ding wan 10 was higher than in Yun wan 8, and the number of DEGs in CC2 was higher than in CC1. These results suggest that the roots of Ding wan 10 with different continuous cropping treatments and Yun wan 8 with CC2 may have been attacked by the pathogens and initiated immunity induced by pathogen molecular correlation patterns. However, there were differences in the degree of infestation between the two pea genotypes.

**Fig. 7:**
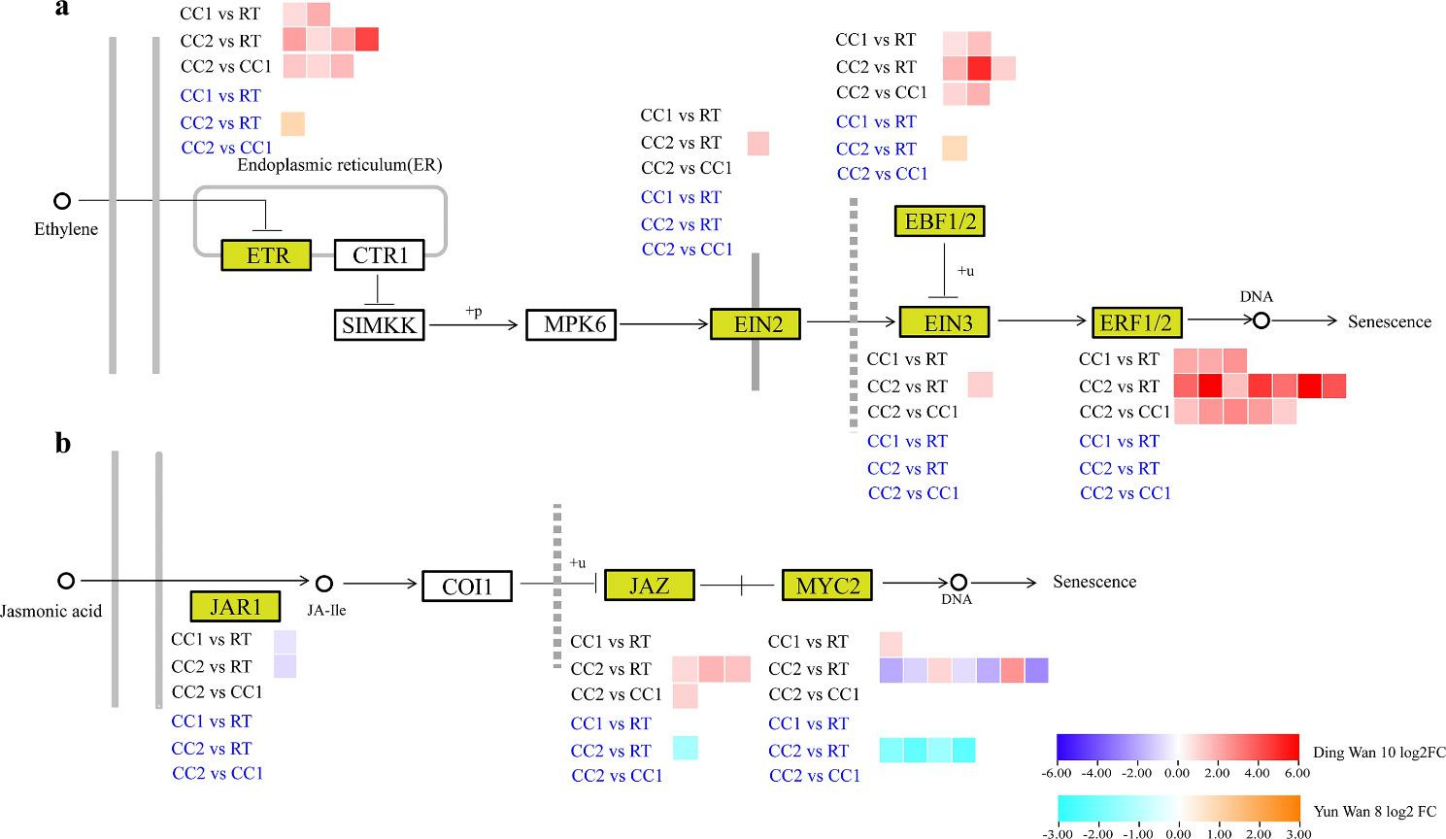
Enrichment of DEGs in plant hormone signal transduction pathway. **a**: Ethylene signal transduction pathway. **b**: Jasmonic acid signaling pathway. **RT**: Rotation treatment. **CC1**: Continuous cropping once treatment. **CC2**: Continuous cropping twice treatment

Interestingly, we observed that *ACS6* was only up-regulated between CC2 and RT in Ding wan 10 **(**Fig. 6**)**, suggesting that ethylene synthesis may be one of the reasons why pea plants are sensitive to continuous cropping. In Ding wan 10, we found that DEGs (*ETR, EBF1/2, ERF1/2*) in the ethylene signaling pathway were up-regulated between treatments (Fig. 7a). However, *EIN2* and *EIN3* were only up-regulated between CC2 and RT. In Yun wan 8, only *ETR* was up-regulated between CC2 and RT (Fig. 7a). In the jasmonic acid signaling pathway of Ding wan 10 (Fig. 7b), *JAZ* and *MYC2* were differentially expressed between the continuous cropping treatment and RT, with *JAZ* up-regulated between CC2 and CC1 and CC2 and RT. In Yun wan 8, *JAZ* and *MYC2* were only down-regulated between CC2 and RT (Fig. 7b). These results indicate that the ethylene and jasmonic acid signal transduction pathways respond to continuous cropping, but the degree of response differs according to the pea genotypes.

Phenylpropanoid biosynthesis and flavonoid biosynthesis are involved in plant immune response. This study, found that most of the genes encoding lignin synthesis were up-regulated in the phenylpropanoid biosynthesis pathway (Fig. S3a-c, Fig. S4a-c). There were more DEGs related to lignin synthesis in CC2 than CC1 of Ding wan 10 (Fig. S3a-c). The changes in DEGs associated with lignin synthesis in Yun wan 8 were similar to those in Ding wan 10, although the differences in the number and variety of DEGs were less pronounced. The changes in DEGs involved in flavonoid biosynthesis were similar to those in lignin (Fig. S5a-c, Fig. S6a-c). These results suggest that pea plants could resist the immune response induced by continuous cropping by increasing their content of flavonoids and lignin.

### Integrative transcriptomic and metabolomic analyses

To clarify the metabolic pathways and metabolites related to pea roots and continuous cropping at the gene and metabolite levels, we analyzed the metabolic pathways co-annotated by DEGs and DAMs. We obtained 32 co-annotated pathways of DEGs and DAMs in CC1 of Ding wan 10 (Fig. S7a), compared to the RT, and 70 co-annotated pathways in CC2 (Fig. S7b). Similarly, we obtained 33 co-annotated pathways of DEGs and DAMs in CC1 of Yun wan 8 (Fig. S8a), compared to the RT, and 56 co-annotated pathways in CC2 (Fig. S8b). When comparing CC1 to CC2 of D10, we obtained 53 co-annotated pathways of DEGs and DAMs (Fig. S7c) and only four co-annotated pathways in CC2 of Yun wan 8 (Fig. S8c).

Plants rely on signal transduction pathways and secondary metabolites to cope with stress. Our analysis revealed several pathways, such as flavonoid biosynthesis, flavone and flavonol biosynthesis, glutathione metabolism, cysteine and methionine metabolism, phenylpropanoid biosynthesis, fatty acid and linoleic acid metabolic pathways were co-annotated in CC1 and RT, CC2 and RT, CC2 and CC1 of Ding Wan 10 (Fig. S7a-c). Additionally, alpha-linolenic acid metabolism, biosynthesis of unsaturated fatty acids, diterpenoid biosynthesis, plant hormone signal transduction, and amino sugar and nucleotide sugar metabolism were co-annotated in CC2 and RT, CC2 and CC1 of Ding wan 10 (Fig. S7b, c). However, sphingolipid metabolism and terpenoid backbone biosynthesis were only co-annotated between CC2 and RT (Fig. S7b). In Yun wan 8, pathways for flavonoid biosynthesis, glutathione metabolism, cysteine and methionine metabolism, starch and sucrose metabolism, and linoleic acid metabolism were co-annotated between CC1 and RT, CC2 and RT (Fig. S8a, b). Glutathione metabolism was also co-annotated between CC2 and CC1 (Fig. S8c). However, alpha-linolenic acid metabolism, phenylpropanoid biosynthesis, terpenoid backbone biosynthesis, plant hormone signal transduction, cutin, suberine and wax biosynthesis, amino sugar and nucleotide sugar metabolism, and fatty acid metabolism were only co-annotated in CC2 (Fig. S8b). Plant hormone signal transduction was also co-annotated between CC2 and CC1 (Fig. S8c). These results indicate that the pathways co-annotated in CC1 and CC2 could be the core metabolic pathways of pea roots in response to continuous cropping. On the other hand, pathways annotated only under CC2 could be unique metabolic pathways of pea roots in response to severe continuous cropping.

### Analysis of soil bacterial diversity and richness under different continuous cropping conditions

**Fig. 8:**
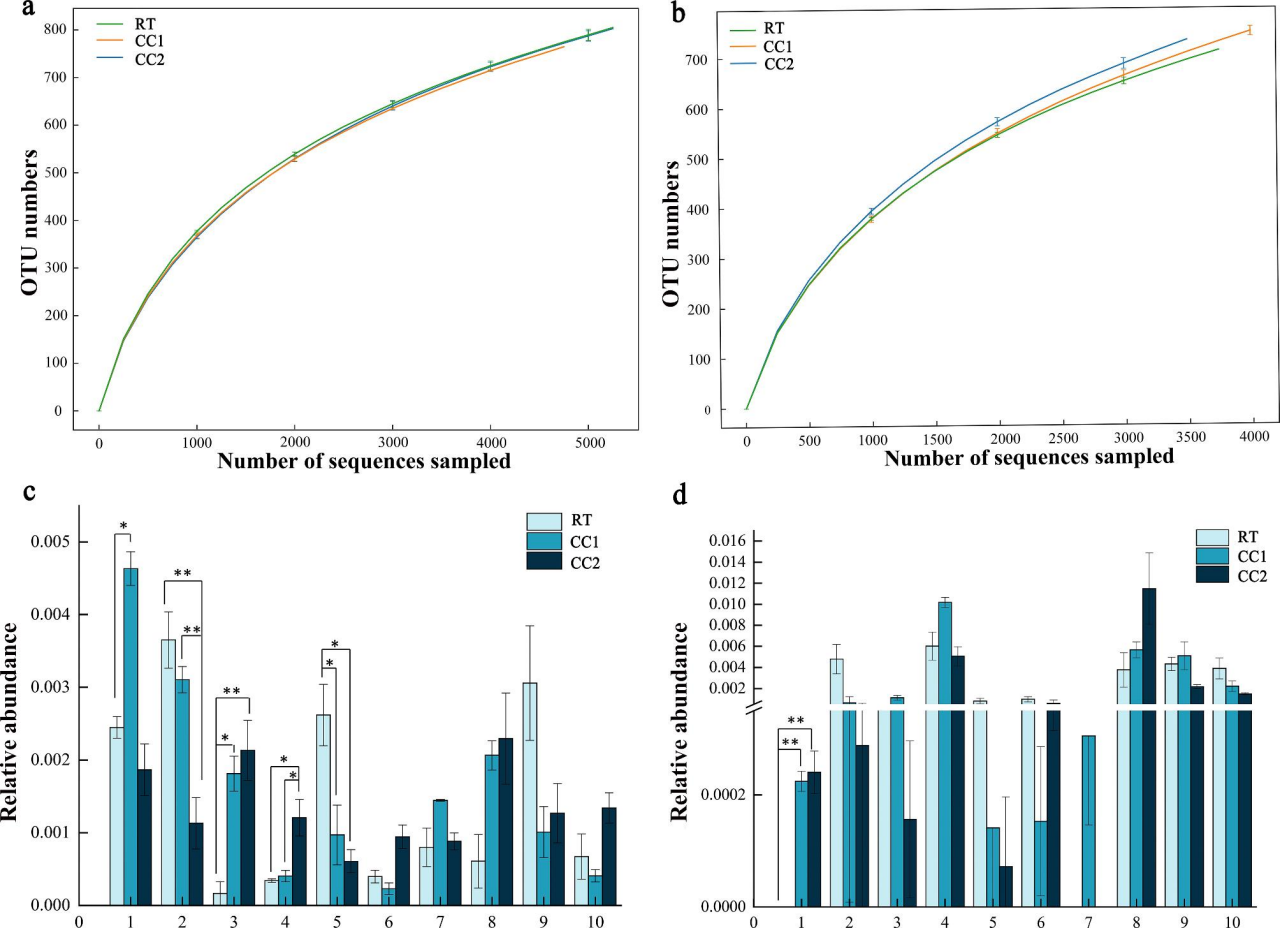
Dilution curves of 16 S rDNA for different genotypes of pea soil bacteria under continuous cropping conditions and analysis of variation between groups. **a** Ding wan 10 dilution curve. **b** Yun wan 8 dilution curve. **c** Inter-group differences based on ANOVA analysis in Ding wan 10. 1 to 10 represent *Chitinophaga, Uncultured_bacteria_o_Azospirillales, Uncultured_bacterium_f_A21b, Uncultured_bacterium_k_Bacteria, Polycyclovorans, Chthonomonas, BIyi10, Ideonella, Flaviaesturariibacte, Ensifer*, respectively. **d** Inter-group differences based on ANOVA analysis in Yun wan 8. 1 to 10 represent *Turicibacter, Bacteroides, Cystobacter, Chitinophaga, Uncultured_bacterium_f_Beijerinckiaceae, Uncultured_bacterium_c_Gitt-GS-136, Bosea, Methylotenera, Ferruginibacter, Uncultured_bacterium_o_C0119*, respectively. **RT**: Rotation treatment. **CC1**: Continuous cropping once treatment. **CC2**: Continuous cropping twice treatment. Calculated *p*-value less than 0.05 were determined to be statistically significant and indicated on graphs. * indicate significant differences at the 0.05 level. ** indicate significant differences at the 0.01 level

Bacterial 16 S rDNA sequencing was used to analyze the cultivation soil of two pea genotypes under different continuous cropping conditions. The dilution curves tended to be flat (Fig. 8a, b), and the coverage ranged from 93.60 to 95.72% (Table S3), indicating that the sequencing volume could cover the vast majority of species in the samples.

Alpha diversity indexes were calculated based on OTU level to quantify the diversity and richness of the microbial community (Table S3). ACE, Chao 1, Shannin and Simpson indices of Ding wan 10 in continuous cropping treatments were lower than in RT, but there was no significant difference among the treatments. The ACE, Chao 1, Shannin and Simpson indices of the continuous cropping treatment in Yun wan 8 were higher than in RT, but there was no significant difference among the treatments. These results showed that the continuous cropping did not affect the diversity and richness of bacteria in pea rhizosphere soil.

### Differences in the distribution of soil bacteria at the phylum level under continuous cropping conditions

We performed 16 S rDNA sequencing on soil samples from Ding wan 10 and Yun wan 8 and identified 26 and 25 phyla at the classification level, respectively. In Ding wan 10, CC2 increased the number of dominant bacteria (> 1%) and reduced the number of rare bacteria (< 0.1%), while common bacteria (0.1-1%) remained unchanged. Similarly, in Yun wan 8, CC2 increased the number of dominant and common bacteria, but reduced the number of rare bacteria. The relative abundance of *Uncultured_bacterium_k_Bacteria* increased with the increase of continuous cropping in Ding wan 10 soil and was significantly higher under CC2 than in RT and CC1. However, the abundance of other bacteria did not change significantly. In contrast, the relative abundance of various bacteria did not change significantly in Yun wan 8.

### Differences in the distribution of soil bacteria at the genus level under continuous cropping conditions

We also analyzed the abundance of soil bacteria at the genus level for the two pea genotypes under different continuous cropping treatments. The analysis of variance among groups on the abundance of bacteria at the genus level in the different treatments revealed that, among the top 10 species with the smallest *p*-value (Fig. 8c, d), four bacteria in Ding wan 10 soil increased or decreased regularly among different treatments. The abundances of *Uncultured_bacterium_o_Azospirillales* and *Polycyclovorans* were lowest under CC2 and highest under RT. The abundance of *Uncultured_bacterium_f_A21b* and *Uncultured_bacterium_k_Bacteria* increased with increasing continuous cropping times, and was highest under CC2 (Fig. 8c). In Yun wan 8 soil, the genus *Turicibacter* increased regularly with increasing continuous cropping times and was highest under CC2 (Fig. 8d). These results suggest that the changes in these bacteria may be related to continuous cropping obstacles.

### Integrative soil bacteria and pea roots metabolomics analyses

**Fig. 9:**
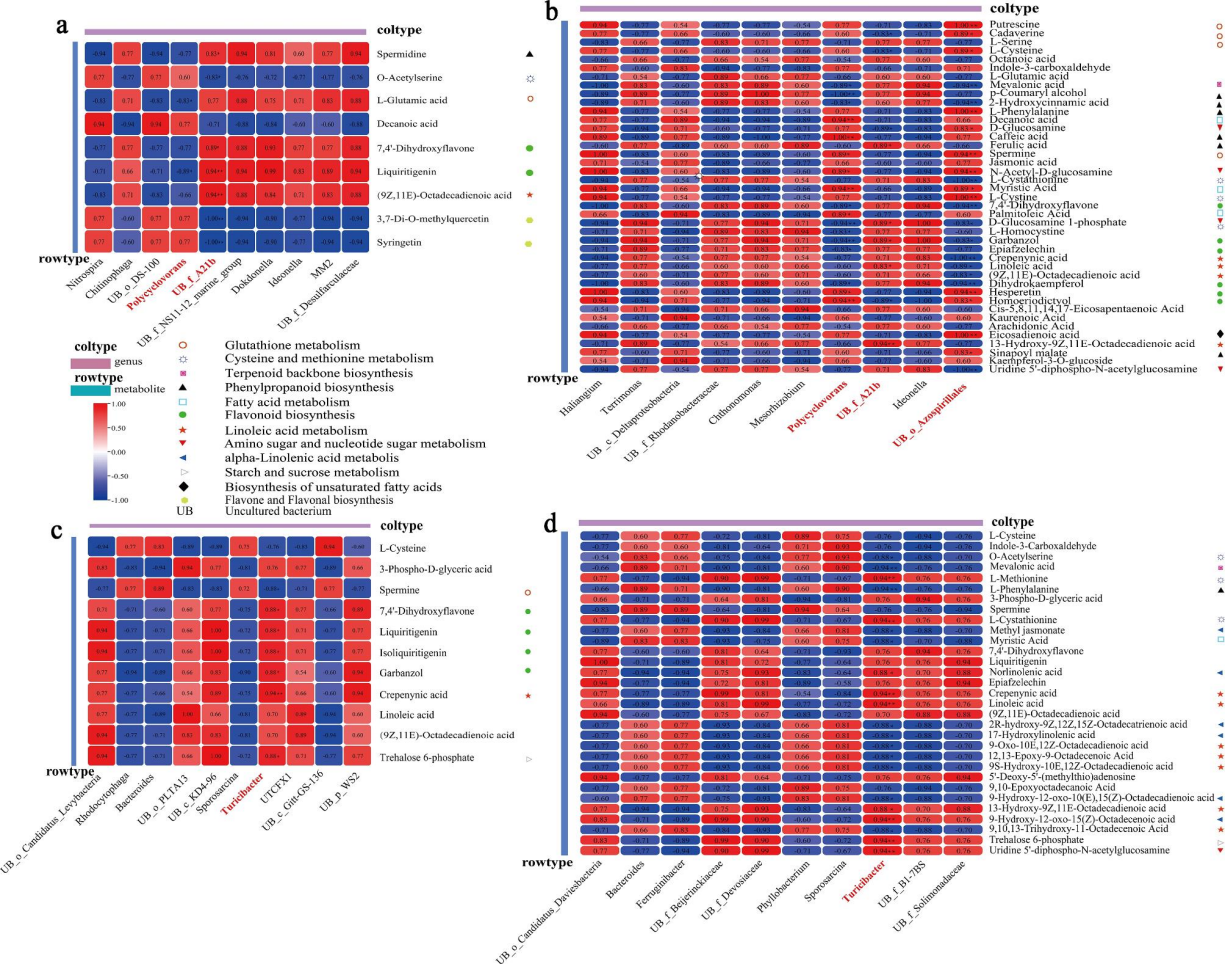
Correlation analysis between metabolites and the top 10 bacteria in relative abundance. **a** and **b** are Ding wan 10. **c** and **d** are Yun wan 8. **RT**: Rotation treatment. **CC1**: Continuous cropping once treatment. **CC2**: Continuous cropping twice treatment. **Red** Positive correlation. **Blue** Negative correlation. Figures in the figure represent the correlation coefficien. * and ** represent significance (*P* < 0.05) and high significance (*P* < 0.01)

Based on the previous experiments, it can be inferred that there is a certain interaction between soil bacteria and the metabolites of pea roots. To explore this relationship, we conducted a correlation analysis between the metabolites involved in the continuous cropping-related metabolic pathways and the top 10 microorganisms with the highest relative abundance of soil bacteria genera. Our analysis found a significant correlation between certain bacteria and some metabolites from pea roots. In Ding wan 10 CC1, soil *Polycyclovorans* bacteria were significantly negatively correlated with liquiritigenin and L-Glutamic acid, two metabolites involved in glutathione metabolism and flavonoid biosynthesis pathways, respectively (Fig. 9a). *Uncultured_bacterium_f_A21b* was significantly related to metabolites involved in phenylpropanoid biosynthesis, flavonoid biosynthesis, linoleic acid metabolism, cysteine and methionine metabolism, and flavone and flavonol metabolism (Fig. 9a). In Ding wan 10 CC2, *Polycyclovorans* were significantly related to metabolites involved in phenylpropanoid biosynthesis, amino sugar and nucleotide sugar metabolism, flavonoid biosynthesis, cysteine and methionine metabolism, fatty acid biosynthesis, terpenoid backbone biosynthesis, and glutathione metabolism pathways (Fig. 9b). *Uncultured_bacterium_f_A21b* was significantly related to metabolites involved in glutathione metabolism, amino sugar and nucleotide sugar metabolism, flavonoid biosynthesis, phenylpropanoid biosynthesis, and linoleic acid metabolism (Fig. 9b). *Uncultured_bacterium_o_Azospirillales* was significantly associated with metabolites involved in glutathione metabolism, terpenoid backbone biosynthesis, phenylpropanoid biosynthesis, amino sugar and nucleotide sugar metabolism, cysteine and methionine metabolism, fatty acid biosynthesis, flavonoid biosynthesis, linoleic acid metabolism, and biosynthesis of unsaturated fatty acids (Fig. 9b). In Yun wan 8, *Turicibacter* in soil from CC1 was significantly associated with glutathione metabolism, linoleic acid metabolism, flavonoid metabolism, and starch and sucrose metabolism (Fig. 9c). *Turicibacter* in soil from CC2 was significantly associated with metabolites involved in cysteine and methionine metabolism, terpenoid backbone biosynthesis, phenylpropanoid biosynthesis, alpha-linolenic acid metabolism, fatty acid biosynthesis, linoleic acid metabolism, starch and sucrose metabolism, and amino sugar and nucleotide sugar metabolism (Fig. 9d). These above results suggest that key bacteria (bacteria with significant changes in relative abundance compared to RT) in soil may influence these metabolic pathways. Additionally, microorganisms with significant changes in relative abundance were found to be significantly related to glutathione metabolism, flavonoid metabolism, and linoleic acid metabolism in CC1 of the two pea genotypes, while microbes with significant changes in relative abundance were significantly related to cysteine and methionine metabolism, phenylpropanoid biosynthesis, amino sugar and nucleotide sugar metabolism, fatty acid biosynthesis, linoleic acid metabolism and terpenoid backbone biosynthesis in CC2 of the two pea genotypes.

## Discussion

Many studies regarding continuous cropping obstacles have been reported, and with the development of modern biotechnology, related studies have been transferred from phenotypic and physiological levels to molecular levels, which is of great value for further revealing the occurrence of continuous cropping obstacles. However, the causes of continuous cropping obstacles are complex. Previous studies have focused on the structure and diversity of soil microbial communities, but there are few studies addressing how crops respond to changes in soil microorganisms [19–22]. There are also studies on the correlation between crop gene level and soil metagenome under continuous cropping, but the key role of metabolites has been ignored [16]. Therefore, based on previous research, this study used 16 S rDNA sequencing technology of soil bacteria, transcriptomics, and metabolomics to analyze and summarize the relationship between pea plants and soil bacteria under continuous cropping conditions, and compared the differences in response to continuous cropping of different pea genotypes. Through duplicate pot experiments, it was found that the growth of Ding wan 10 was inhibited under continuous cropping conditions, and the phenotypic variation was particularly obvious under CC2. There was no significant change in Yun wan 8 under CC1, but its growth was significantly inhibited under CC2. This indicated that pea plants already had severe continuous cropping obstacles under CC2, and that the two pea genotypes had different tolerances to continuous cropping.

The metabolome forms the biochemical basis of the plant phenotype, and the remodeling of the metabolome under stress reflects the response and defense of plants to stress to a great extent [23–25]. Metabolomics revealed that potential autotoxin species in pea roots were abundant, and the level of autotoxins increased with increasing times of continuous cropping. Among them, the autotoxicity of cinnamic acid on pea plants has been demonstrated [26]. However, the validation method relies on the artificial addition of chemical reagents. It needs to be further verified whether the autotoxin synthesized by the pea plants negatively regulates it using molecular techniques in the future. Autotoxins secreted by plant roots are believed to cause continuous cropping obstacles by producing autotoxicity and affecting soil microbial community structure [27–29]. However, it is still necessary to investigate whether the concentration of autotoxins in the soil causes autotoxicity and the mechanisms of regulation of microbial community structure by autotoxins.

This study found that with increasing continuous cropping times, the number of differential metabolites in pea roots increased, and the metabolic pathways involved increased. This means that pea roots adapt to changes in the surrounding environment by changing the composition and content of metabolites. Flavonoid biosynthesis and the linoleic acid metabolism pathway were significantly enriched under different continuous cropping treatments [30, 31]. These metabolic pathways play a key role in plants’ immune response, suggesting that pea roots response to continuous cropping is achieved by regulating metabolic pathways associated with the immune system.

We compared the expression of pea root genes under each treatment using high-throughput sequencing. It was found that a large number of DEGs in the roots of the two pea genotypes under different continuous cropping treatments were associated with plant-pathogen interactions. Wyrsch et al. discovered that *FLS2* expression could trigger an immune response in roots [32]. *FLS2* is induced by flg22 to bind to *BAK1*, forming a heterologous aggregate that induces a signaling cascade including reactive oxygen species generation, calcium signaling, MAPK phosphorylation, and gene transcription, ultimately leading to defense response [33–35]. In this study, *FLS2*, *BAK1*, *CaMCML*, and *MKK4/5* were activated under CC1 treatment in Ding wan 10 and CC2 treatment in Yun wan 8. This indicates that under continuous cropping conditions, the pea roots initiate an immune response that triggers calcium signaling and MAPK signaling cascades, and activates the transcription factor (*WRKY22, WRKY33*) involved in defense gene expression. The number of DEGs involved in calcium signaling and MAPK signaling cascade responses increased and most of them were up-regulated under CC2 treatment in Ding wan 10. This suggests that the strong response of these genes under CC2 treatment may be responsible for the inhibition of pea plant growth.

The phenylpropane biosynthetic pathway and flavonoid biosynthetic pathway are involved in the immune and defense responses of plants [31, 36, 37]. Among them, the accumulation of lignin and flavonoids can resist the invasion of pathogenic bacteria [38–41]. In this study, continuous cropping activated the expression of key genes involved in lignin and flavonoid biosynthesis, especially under CC2 treatment. These results indicate that lignin and flavonoid synthesis was the main defense measures of pea roots against immune response. Furthermore, it was confirmed that the ethylene and jasmonic acid signaling pathways were important pathways for plant pattern-triggered immune (PTI) responses, and that increased levels of ethylene and jasmonic acid inhibit plant growth [42, 43]. In this study, key genes involved in the ethylene and jasmonate signaling pathways (*ETR*, *EIN2*, *EIN3*, *EBF1/2*, *ERF1/2*) were found to be up-regulated in CC2 of Ding wan 10. Moreover, *ACS6* [44, 45] involved in ethylene synthesis was up-regulated. This indicates that the phytohormone signaling pathway may be a specific response pathway under severe continuous cropping treatment in peas and a possible reason for the differences in tolerance to continuous cropping in different pea genotypes.

A combined transcriptome and metabolome analysis showed that the metabolic pathways related to antioxidant synthesis (flavonoid biosynthesis, cysteine and methionine metabolism, and glutathione metabolism pathway) were co-annotated in different treatments of the two pea genotypes. This is similar to a study by Huang et al. on continuous cropping obstacles in sugar beet [46]. In addition, linoleic acid metabolism was the co-annotated metabolic pathway in different continuous cropping treatments. It has been reported that linoleic acid metabolism was a typical PTI response [30], indicating that continuous cropping triggered an immune response in pea roots and caused oxidative stress. Furthermore, we found that alpha-linolenic acid metabolism was co-annotated under CC2 of the two pea genotypes, which is similar to linoleic acid metabolism [47]. We speculate that although the metabolism of linoleic acid and alpha-linolenic acid are both typical PTI reactions, the conditions under which their metabolisms are initiated may be related to the degree of continuous cropping, and this needs to be verified in future studies. Plant hormone signal transduction, fatty acid biosynthesis, terpenoid backbone biosynthesis, and amino sugar and nucleotide sugar metabolism pathways are related to continuous cropping disorders in peanut, soybean and melon, respectively [16, 48, 49]. In this study, these metabolic pathways were co-annotated under CC2 in only two pea genotypes, indicating that alterations in these metabolic pathways are key to the stunted growth of pea plants and a specific response mechanism made by the pea roots under severe continuous cropping.

In this experiment, the cultivation substrates differed for each treatment and the remaining management measures were the same. Therefore, the cause of the change in plant phenotypes could only be due to differences in the soil. The content of soil nutrient elements in each treatment before planting was detected (Table S5), and combined with the change in pea phenotypes, it was revealed that soil nutrient elements were not the reason for the growth of pea plants. The presence of autotoxins in the soil is also an important factor in continuous cropping obstacles [28, 50]. However, in most cases, the concentration of autotoxins in the soil is lower than the concentration that causes autotoxic stress in plants, and microorganisms easily degrade the autotoxins in the soil [51, 52]. Some studies have reported that pea autotoxins are mainly secreted during the vegetative growth stage [53], whereas the soil in this experiment was left fallow for one month at the end of the pea growth period and then left for one month until it dried naturally. Therefore, autotoxins are not the main cause of growth inhibition in pea plants. These results indicate that soil microorganisms play an irreplaceable role in the occurrence of continuous cropping obstacles.

Soil microorganisms play a crucial role in crop growth, development, and health [54–56], and have garnered considerable attention in the study of continuous cropping obstacles. In a study on the continuous cropping of peanuts, Li et al. found that bacterial suspensions obtained from continuous cropping soils significantly inhibited the growth of peanut plants, indicating the importance of soil bacteria in continuous cropping [16]. Based on previous research, we analyzed bacterial communities in rhizosphere soils and found that continuous cropping did not affect bacterial diversity in pea rhizosphere soil, which was consistent with the results of a study by Yuan et al. in soybeans [10], but differed from the findings of Li et al. in peanuts [16]. This difference may be attributed to the crop type and continuous cropping duration. However, continuous cropping significantly altered the relative abundance of four types of bacteria in Ding wan 10 soil and only one in Yun wan 8 soil. The variation in microbial species in the soil of the two pea genotypes may be attributed to differences in the pea genotypes, leading to different root secretions that recruit distinct bacterial communities to the rhizosphere, resulting in variations in bacterial communities in the soil of the two pea genotypes [57–60]. Additionally, continuous cropping led to in the repeated release of the same type of root exudates into the soil, which stimulated the colonization of certain microorganisms in the pea rhizosphere and increased their relative abundance, ultimately triggering an immune response in the pea roots. Future studies should confirm the vital role of these bacteria in continuous cropping obstacles of peas through the isolation, purification, and biological verification of the strains. Furthermore, the disparity in the number of significantly changing bacteria in the rhizosphere soil of the two pea genotypes may be the key to the difference in their sensitivity to continuous cropping.

**Fig. 10:**
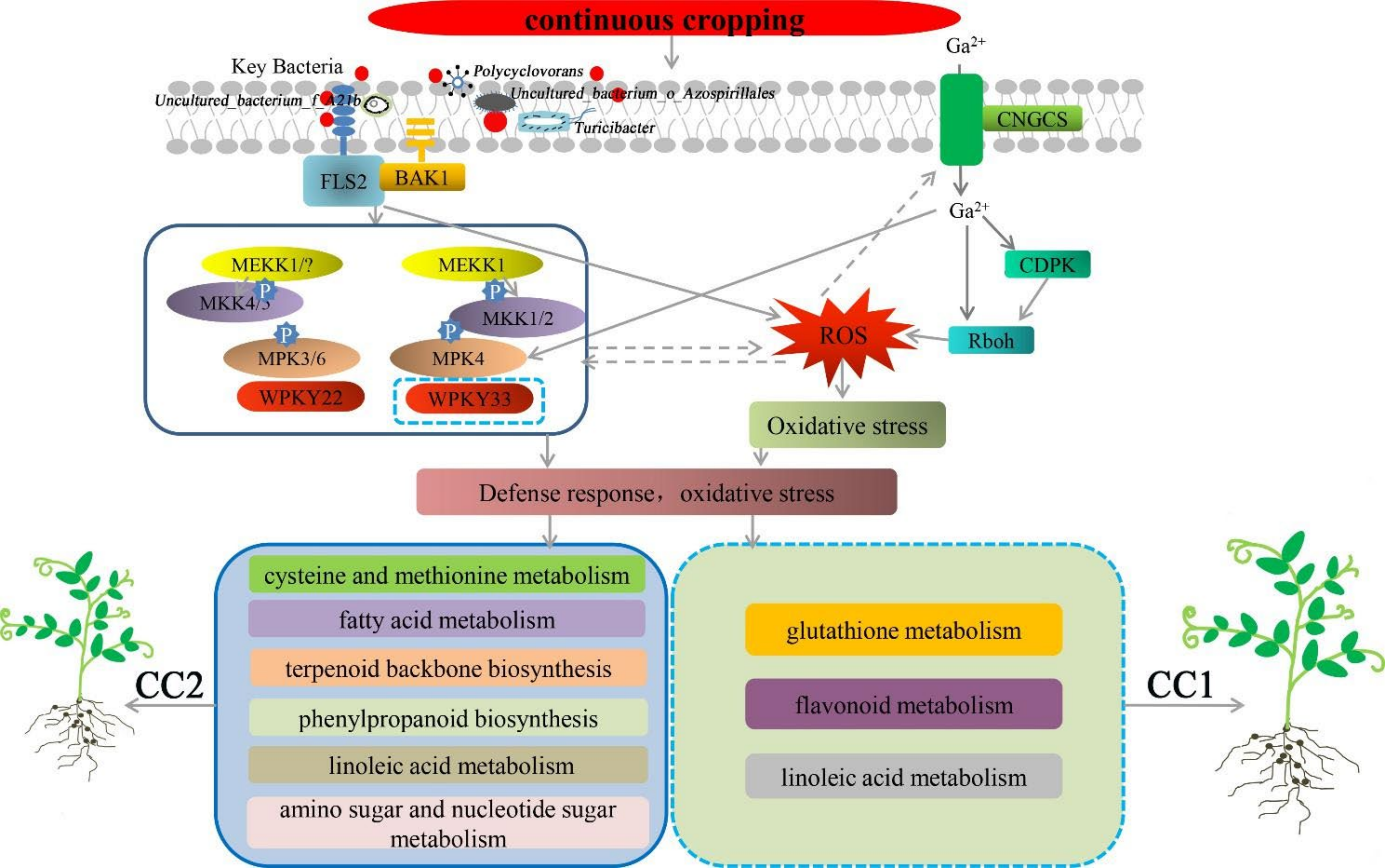
Schematic presentation of response mechanism of pea roots to continuous cropping obstacles

The growth of crops is influenced by microorganisms, such as bacteria in the soil, while the levels of their metabolites primarily regulate changes in crop phenotypes. Therefore, understanding the relationship between soil bacteria and metabolites related to continuous cropping is crucial for determining the occurrence of pea continuous cropping obstacles. This study, found that the significantly altered bacteria in CC1 were significantly associated with immune responses in pea roots and antioxidant synthesis pathways, such as linoleic acid metabolism, glutathione metabolism, and flavonoid metabolism. These results suggest that key soil bacteria may induce mild immune responses in pea roots under mild continuous cropping conditions, leading to oxidative stress. This response may be a common pathway for both pea genotypes in response to mild continuous cropping (Fig. 10). The number of root metabolic pathways significantly associated with bacteria increased under CC2 treatment, involving cysteine and methionine metabolism, fatty acid metabolism, phenylpropanoid biosynthesis, terpenoid backbone biosynthesis, linoleic acid metabolism, and amino sugar and nucleotide sugar metabolism pathways. These metabolic pathways are specific response strategies of pea plants to severe continuous cropping environments, and the CC1 treatment did not induce pea plants to modulate these metabolic pathways. Moreover, these metabolic pathways are involved in plants’ oxidative stress and defense responses. For instance, cysteine and methionine metabolism are related to antioxidant biosynthesis [49], while fatty acids are essential components of cell membranes and important for remodeling membrane fluidity, affecting plant resistance to adversity stresses [61, 62]. This suggests the severe continuous cropping environment may have caused oxidative damage to pea roots and induced membrane lipid peroxidation. Linoleic acid metabolism can affect callose accumulation [30, 47], which is biosynthesized by UDP glucose, a downstream amino and nucleotide sugar metabolism [63–65]. Under stress, callose deposits instantaneously and reversibly on specific cell walls. The metabolism of amino sugars and nucleoside sugars is related to maintaining and repairing the cell wall [66], and lignin synthesis depends on the phenylpropane metabolic pathway. The accumulation of lignin and callose can enhance the mechanical strength of plant cell walls and block the diffusion channel between cells. Moreover, terpenoids can improve plant stress resistance [67–69]. This indicates that the pea root system responds to severe continuous cropping environments by regulating multiple defense-related metabolic pathways (Fig. 10). These results further illustrate the relationship between changes in bacterial abundance in soil and phenotypic changes in pea plants under continuous cropping conditions. These key bacterial and metabolic pathways warrant further investigation in future studies of continuous cropping obstacles in peas.

## Conclusion

In this study, phenotypic observations revealed that continuous cropping inhibited the growth of pea plants. Transcriptomics and metabolomics were used to analyze changes in the pea root system at the gene and metabolic levels, and the number of DEGs and DAMs increased with increasing continuous cropping times. Common changes in genes and metabolites were induced by different continuous cropping treatments in flavonoid metabolism, glutathione metabolism, linoleic acid metabolism, and other metabolic pathways in pea roots. However, severe continuous cropping induced common changes in genes and metabolites in plant hormone signal transduction, fatty acid biosynthesis, terpenoid backbone biosynthesis, and amino sugar and nucleotide sugar metabolism pathways in pea roots.

To investigate the effects of soil bacteria on the growth of pea plants, 16 S rDNA technology was used to analyze changes in the soil bacterial community structure. Soil bacterial diversity remained unchanged after continuous cropping, while the relative abundance of bacteria changed. Under mild continuous cropping conditions, bacteria with significant changes in relative abundance affected flavonoid metabolism, glutathione metabolism, and linoleic acid metabolism in pea roots. With increasing continuous cropping times, these bacteria affected the growth of pea plants through cysteine and methionine metabolism, fatty acid metabolism, phenylpropanoid biosynthesis, terpenoid backbone biosynthesis, linoleic acid metabolism, and amino sugar and nucleotide sugar metabolism. Furthermore, the two pea genotypes exhibited different sensitivities to continuous cropping, with the roots of sensitive peas showing more DEGs, DAMs, and bacteria. The results of this study provide a deeper understanding of the defense strategies of pea roots against continuous cropping environments and offer new insights into the occurrence of continuous cropping obstacles in peas.

## Methods

### Acquisition and treatment of test soil

The field experiments were performed at the Yuhe village, Anding District, Dingxi City, Gansu Province, an arid and semi-arid rain fed agricultural area in western China. This area belongs to loess hilly and gully area, with a ground span of N35° 17ʹ54ʺ to 36° 02ʹ40ʺ, E104° 12ʹ48ʺ to 105° 01ʹ06ʺ. The average annual sunshine is 2500.1 h; the average annual temperature is 6.3 ℃; the frost-free period is 141 days; the normal annual precipitation is about 400 mm, mostly in autumn; and evaporation is as high as 1500 mm. The trial site had not been planted with legume crops during the previous 10 years. Two pea genotypes seeds were selected for the trial: Ding wan 10 (Common leaf pea) and Yun wan 8 (Semi-leafless pea), both provided by the Dingxi Institute of Agricultural Science in Gansu Province, China.

The field experiments were conducted over two years (2020 and 2021), with the preceding crop being corn in 2019. The site was divided into 18 plots (each 18 m^2^), which were used to cultivate two types of crops: (1) Oil flax was planted in 12 plots, and (2) pea crops were planted in six plots (three plots for each genotype). In the second year of the field experiments (2021), three treatments were applied: (1) Six plots that had grown oil flax in 2020 were used to grow potatoes; (2) Six plots that had grown oil flax in 2020 were used to grow peas (three plots for each genotype); (3) Six plots where peas were planted in 2020 continued to grow peas. Planting, weeding, and harvesting were carried out manually, with planting taking place in April and harvesting in July. The soil pH and the content of soil nutrient elements are presented in Table S5.

### Greenhouse pot experiments simulating continuous pea cropping

On August 18, 2021, soil samples were collected from unplanted pea soil, soil planted with different pea genotypes for one year, and soil planted with different pea genotypes for two years. Each treatment had three replicate plots, and 25 kg (0–20 cm layer) soil samples were randomly collected from each plot and brought back to the laboratory. After removing visible plant residues from the soil, the samples were allowed to dry naturally for the greenhouse pot experiment. The pot experiment was a two-factor experiment, including pea genotypes and different continuous cropping times. Each pea genotype had three treatments: RT, CC1, and CC2, with nine biological replicates per treatment (three biological replicates per plot × three plots = nine). Four kilograms of soil per pot, and 15 surface sterilized pea seeds were sown in each pot, for 54 pots (two genotypes × three treatments × nine replicates = 54 pots). The pot experiment was repeated twice on August 24 and September 22, 2021, to the ensure reproducibility and authenticity of the experiment.

After 16 days of cultivation, plants were carefully removed from the pots and rhizosphere samples were collected by brushing away the soil adhering to the roots. At the same time, the roots of pea plants were cleaned with sterile water. The collected soil and root samples were frozen in liquid nitrogen and stored at − 80 °C. For subsequent analysis, three soil and pea root samples were collected for each treatment. In the following text, we refer to this experiment’s three treatments RT, CC1, CC2.

### Determination of shoot height, root length, and shoot (or root) fresh weight

Root and shoot were separated and the water on the root surface was gently drained, and the shoot (or root) fresh weight were measured immediately. Meanwhile, plant height and root length were measured.

### Metabolite extraction and metabolomics analysis

Pea root metabolites were determined by liquid chromatography with tandem mass spectrometry (LC-MS/MS). Samples were ground after freeze-dried and ground and dissolved in 1.2 mL methanol and 2 µL internal standard (2-Chloro-L-phenylalanine), and vortexed once (30 s) every 30 min, a total of six times. Then, the samples were sonicated for 5 min (incubated in ice water) and incubated for 1 h at − 20 °C to precipitate the proteins. Thereafter, the samples were centrifuged at 4 °C for 15 min (12,000 × g). After centrifugation, the supernatant was collected, filtered through a microporous membrane, and stored in an injection flask for LC-MS/MS analysis. LC-MS/MS analysis was performed on a Waters ACQUITY UPLC I-Class PLUS system. MS raw data were collected using MassLynx (v.4.2, Waters) software. Data processing was carried out using Progenesis QI software. The METLIN database and Biomark self-built library identification database were used for peak annotation identification. DAMs were extracted based on the values retrieved by variable importance in the projection (VIP > 1), T-test (*P* < 0.05), and |Fold Change (FC)| > 1. Metabolite analysis was performed using BMK Cloud (http://www.biocloud.net/).

### Transcriptomics sequencing and bioinformatics analysis

RNA was extracted from roots tissues of different treatments, with three biological replicates per treatment. RNAs were extracted using the DP441 test kit. RNA concentration and integrity were detected using Nanodrop 2000 and Agient 2100, respectively. mRNA was enriched and randomly interrupted. Using mRNA as a template, the first strand of cDNA was synthesized with random hexamer primers. The second strand of cDNA was synthesized through dNTPs, RNase H, and DNA polymerase. cDNA was purified using AMPure XP beads, and end repair and linkage sequencing linkers were performed. Fragment size selection was performed with AMPure XP beads, and cDNA libraries were obtained by PCR enrichment. The effective concentration of the library (Library effective concentration > 2 nm) was accurately quantified using Q-PCR. cDNA libraries were sequenced using the Illumina platform.

The raw RNA reads were filtered to obtain high-quality clean reads by removing adaptor sequences and low-quality bases. The clean reads were aligned to the pea reference genome using HISAT2 software (https://urgi.versailles.inra.fr/Species/Pisum). StringTie was used to assemble the transcript and the gene expression was calculated.

The Deseq 21.6.3 software package was used to select DEGs, and FC and False Discovery Rate (FDR) were used as screening criteria. The FDR is obtained by correcting the difference significance *p*-value; specifically |FC| > 1.5 and FDR < 0.05. GO and KEGG pathway enrichment analysis for differentially expressed genes were implemented using the BMK Cloud platform (http://www.biocloud.net/). The GO term enrichment analysis of DEGs was performed using the GOseq R package [70]. Statistical enrichment of DEGs in the KEGG [71] pathway was performed using KOBAS [72].

### Microbial DNA extraction, 16 S rDNA gene sequencing, and data analysis

The extraction of nucleic acids was carried out using a DNA test kit (Model: DP812). The nucleic acid concentration was detected using a microplate reader (Model: Synergyhtx). The DNA was amplified by 16 S rDNA primers the full-length of 27 F (5’- AGRGTTTGATYNTGGCTCAG-3’) and 1492R (5’-TASGGHTACCTTGTTASGACTT-3’) [73, 74], and complete the library construction. The library concentration and size were detected using Qubit and Agilent 2100, respectively, and then sequenced on a sequel II (Pacbio, USA) sequencer.

The offline data of PacBio Sequel was in Bam format, and Circular Consensus Sequencing (CCS) files were exported through smrtlink analysis software; barcode identification was performed on CCS sequences, length filtering was performed, chimeras were removed, and Effective CCS was obtained. The UCHIME algorithm (v8.1) was used to detect and remove chimera sequences to obtain clean reads. The unique sequence set was classified into OTUs based on a 97% threshold identity using USEARCH (v10.0) [75]. Subsequently, taxonomy annotation of the OTUs was performed based on the Naive Bayes classifier in QIIME2 [76] using the SILVA database [77]. Alpha diversity was calculated using QIIME2. Group comparisons were performed using ANOVA analysis of variance with the BH-FDR multiple comparisons test.

### Integrated Analysis of Transcriptome and Metabolome

The correlation between DEGs and DAMs was analyzed by Pearson (PCC > |0.8|, *P <* 0.05). All DEGs and DAMs were simultaneously mapped to the KEGG pathway database to determine the common metabolic pathways in which DEGs and DAMs participated.

### Integrated Analysis of metabolome and bacteria

Correlation analyses between soil bacteria and DAMs were performed using Spearman.

### Statistical analysis

Shoot height, root length, shoot (or root) fresh weight, soil pH, and soil nutrient content measurement and analysis were performed with at least three biological replicates. Statistical analysis, tabulation, and plotting were carried out using SPSS22.0, Microsoft Excel 2010 and Origin 9.

## Electronic supplementary material

Below is the link to the electronic supplementary material.

Supplementary Material 1

Supplementary Material 2

Supplementary Material 3

Supplementary Material 4

Supplementary Material 5

Supplementary Material 6

Supplementary Material 7

Supplementary Material 8

Supplementary Material 9

Supplementary Material 10

Supplementary Material 11

Supplementary Material 12

Supplementary Material 13

## Data Availability

Transcriptome sequence data and microbial sequence data used in the current study are available in the NCBI Sequence Read Archive under the accession number BioProject PRJNA889933 and PRJNA891063, respectively (https://www.ncbi.nlm.nih.gov/bioproject/).

## Abbreviations

RT: Rotation treatment
CC1: Continuous cropping once treatment
CC2: Continuous cropping twice treatment
DEGs: Differentially expressed genes
DAMs: Differentially accumulated metabolites
GO: Gene Ontology
KEGG: Kyoto Encyclopedia of Genes and Genomes
FDR: False Discovery Rate
VIP: Variable importance in the projection
FC: Fold Change
PCC: Pearson’s correlation coefficient
