# Supplementary figures and images for: New insights into the occurrence of continuous cropping obstacles in pea (Pisum sativum L.) from soil bacterial communities, root metabolism and gene transcription
Source: BMC Plant Biol. 2023 Apr 28;23:226. doi: 10.1186/s12870-023-04225-8 (PMC10141910; doi:10.1186/s12870-023-04225-8)

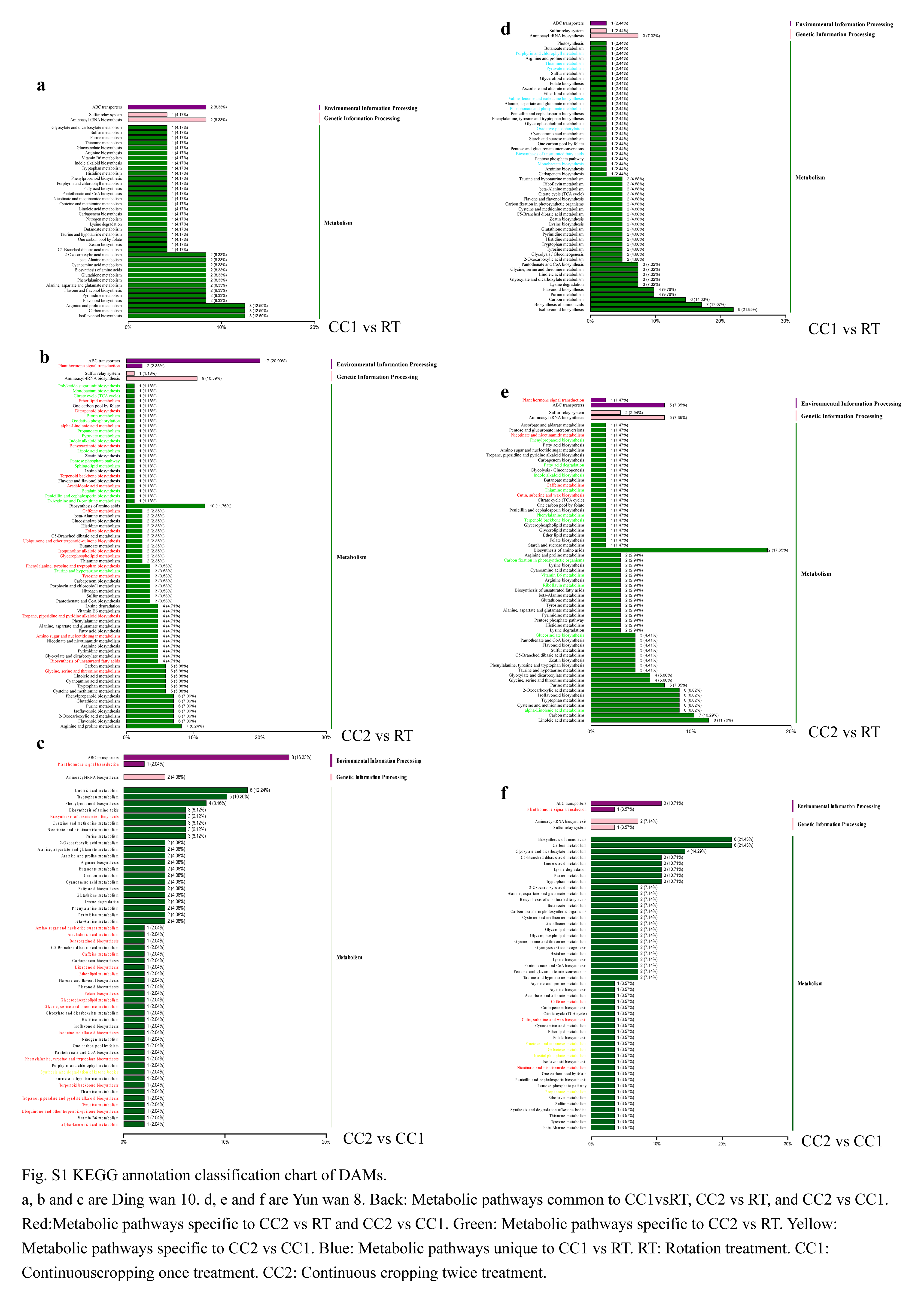

Supplement: Supplementary file 6 — Supplementary Material 6 [file 12870_2023_4225_MOESM6_ESM.tif]

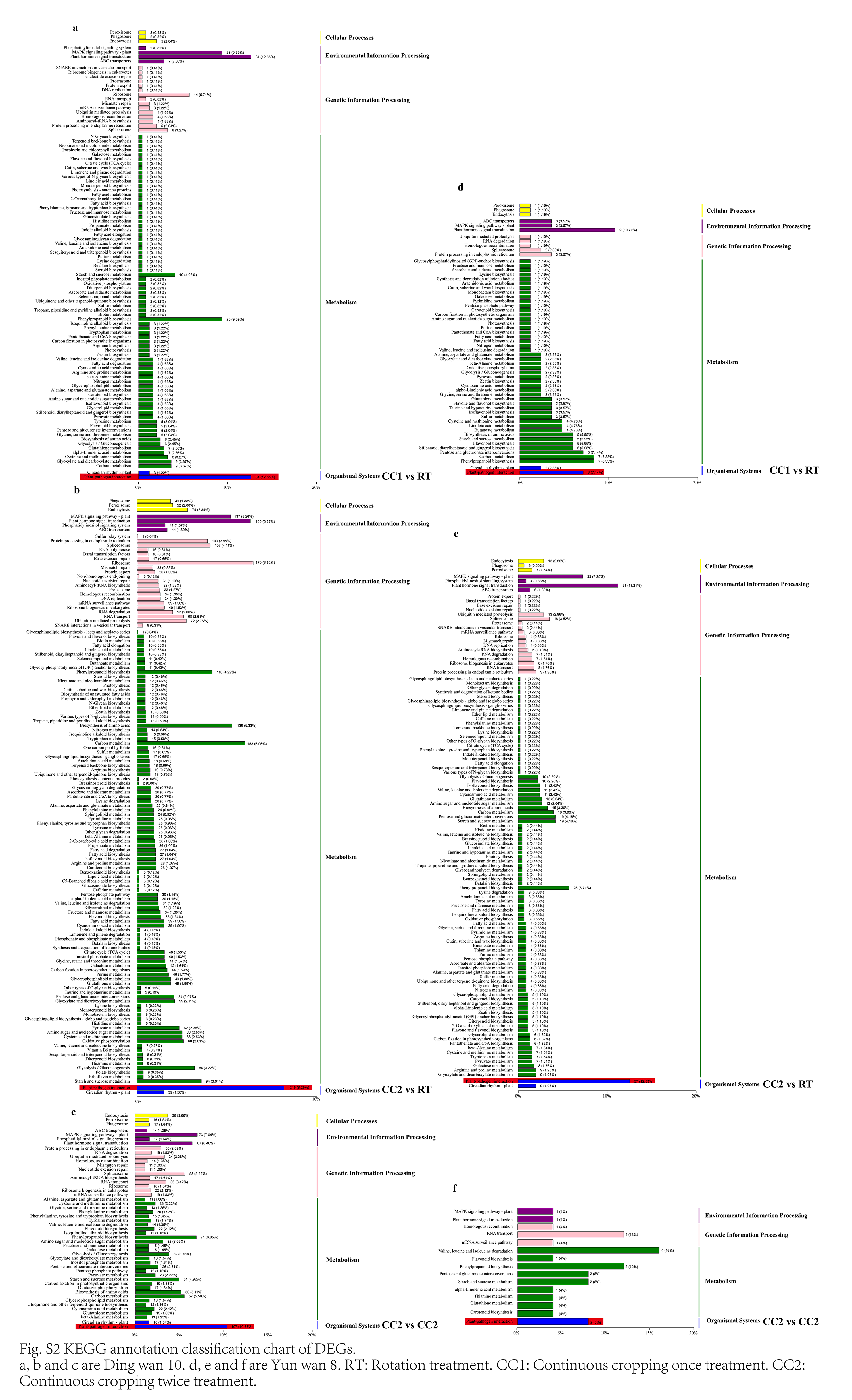

Supplement: Supplementary file 7 — Supplementary Material 7 [file 12870_2023_4225_MOESM7_ESM.tif]

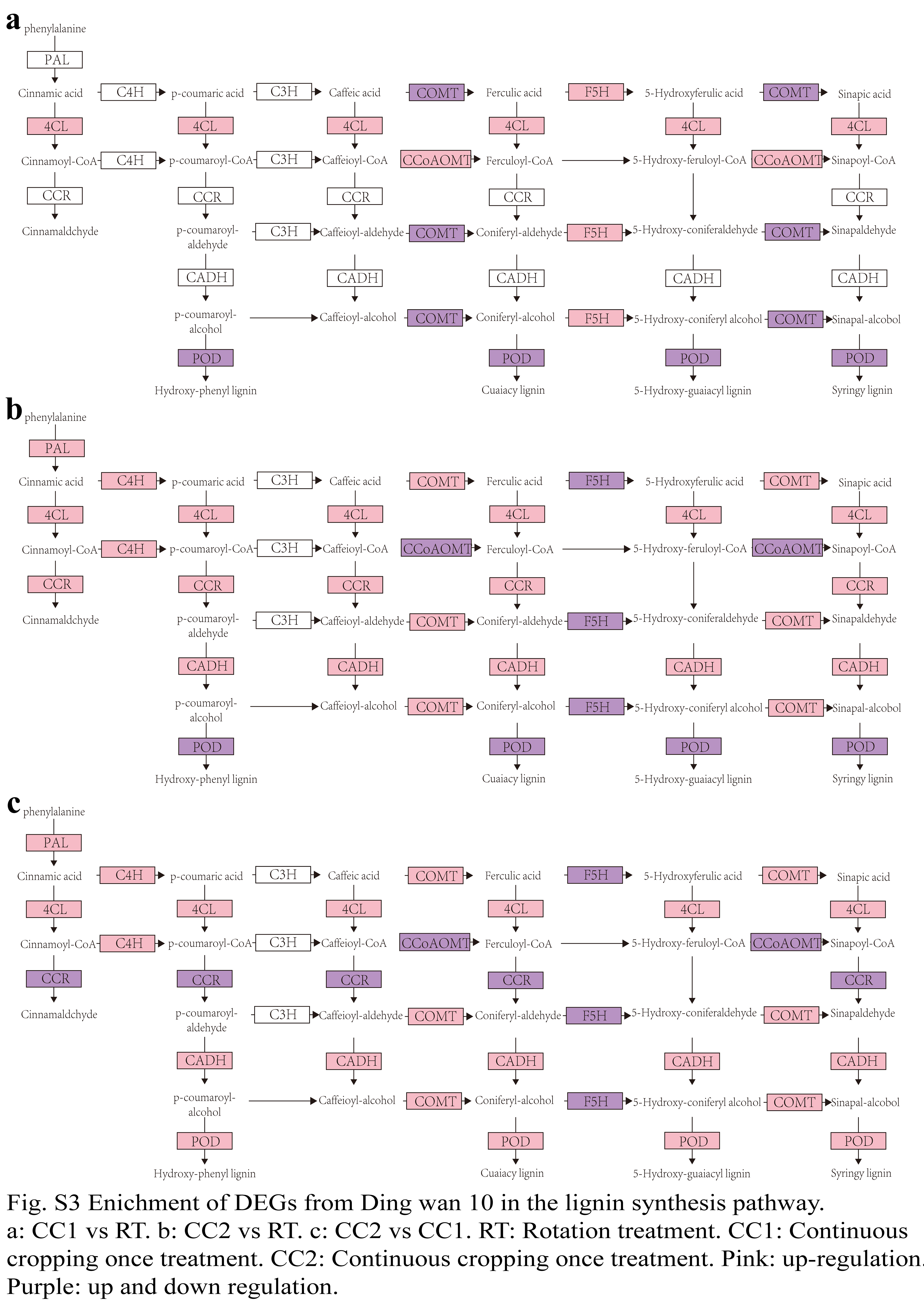

Supplement: Supplementary file 8 — Supplementary Material 8 [file 12870_2023_4225_MOESM8_ESM.tif]

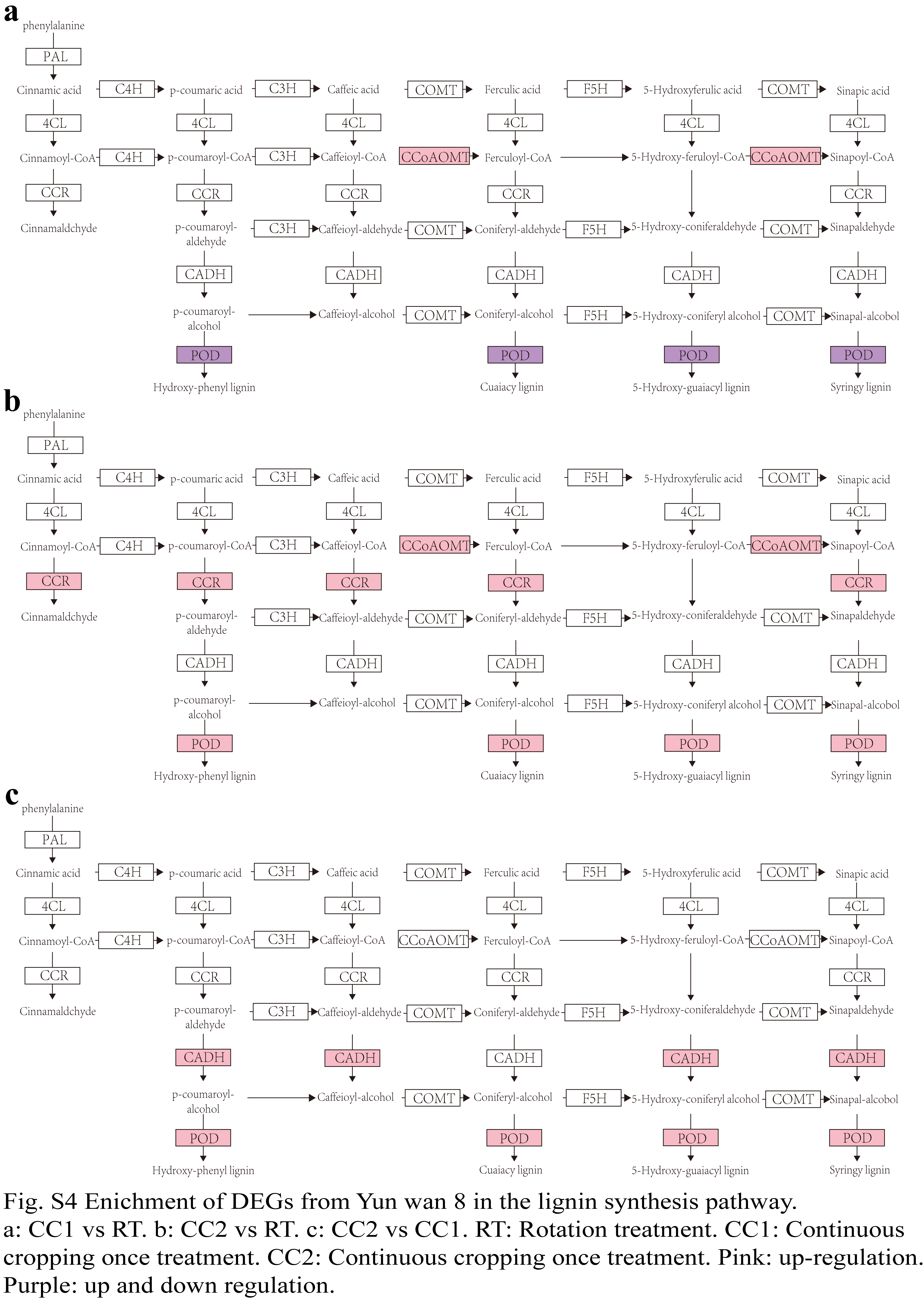

Supplement: Supplementary file 9 — Supplementary Material 9 [file 12870_2023_4225_MOESM9_ESM.tif]

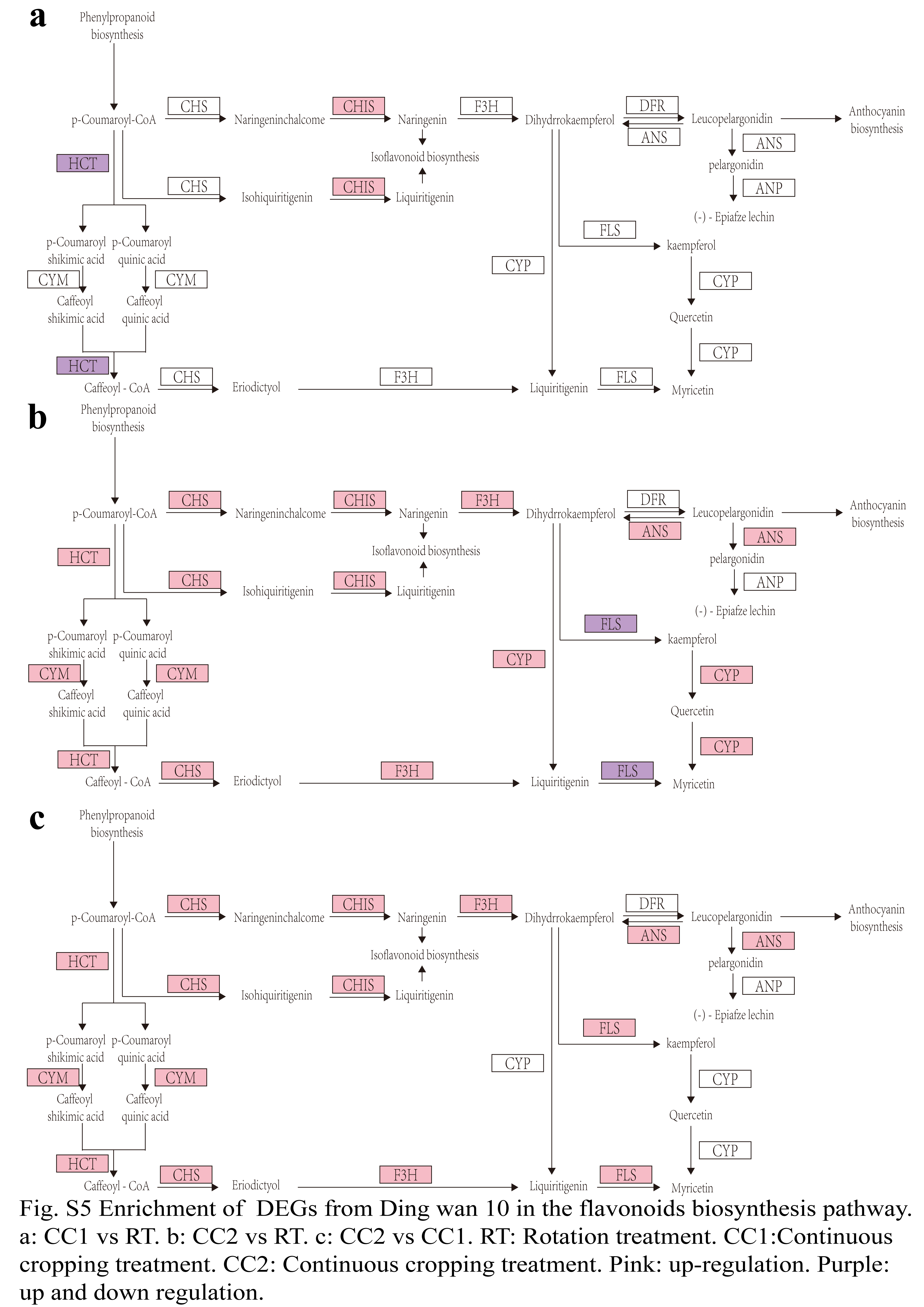

Supplement: Supplementary file 10 — Supplementary Material 10 [file 12870_2023_4225_MOESM10_ESM.tif]

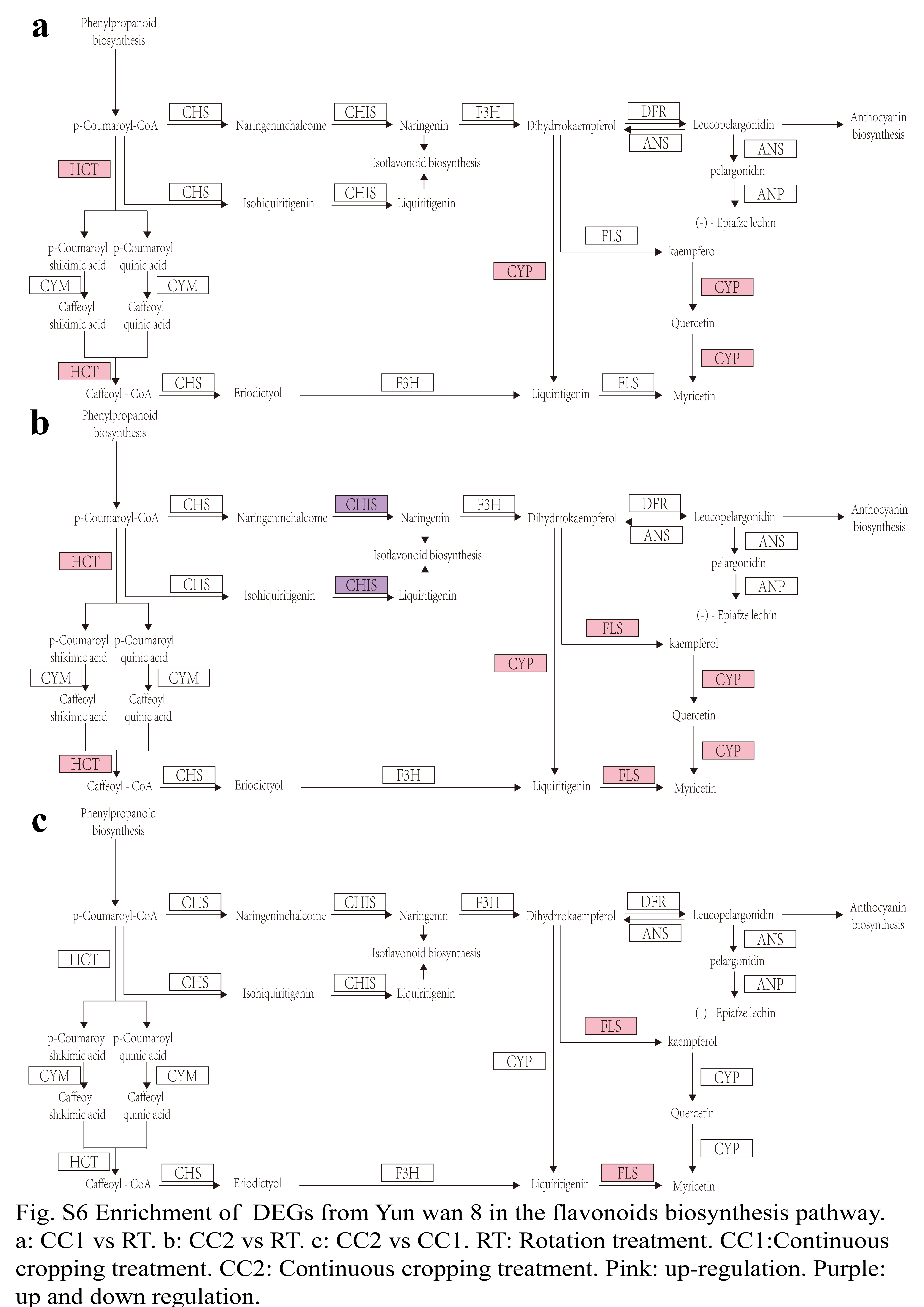

Supplement: Supplementary file 11 — Supplementary Material 11 [file 12870_2023_4225_MOESM11_ESM.tif]

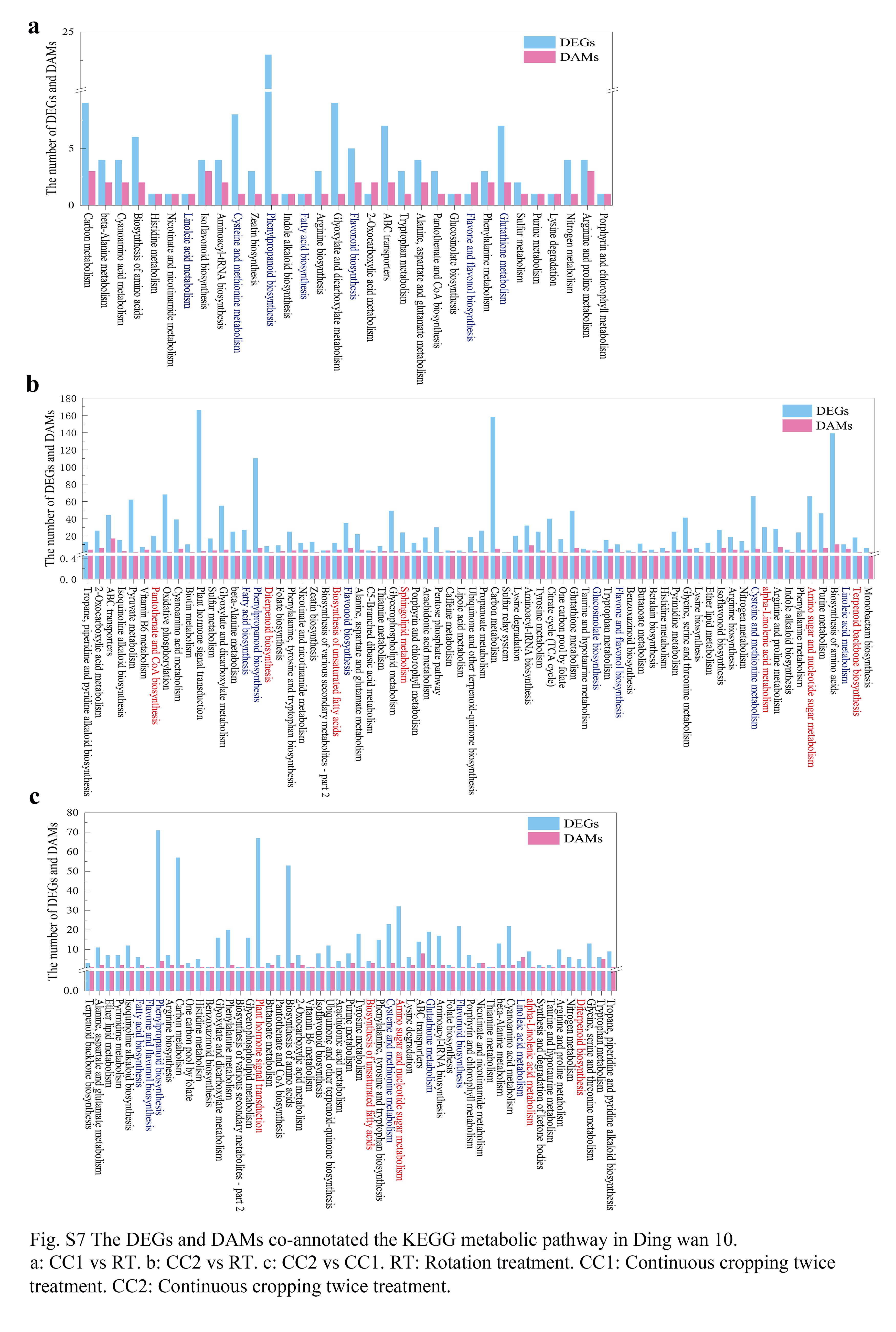

Supplement: Supplementary file 12 — Supplementary Material 12 [file 12870_2023_4225_MOESM12_ESM.tif]

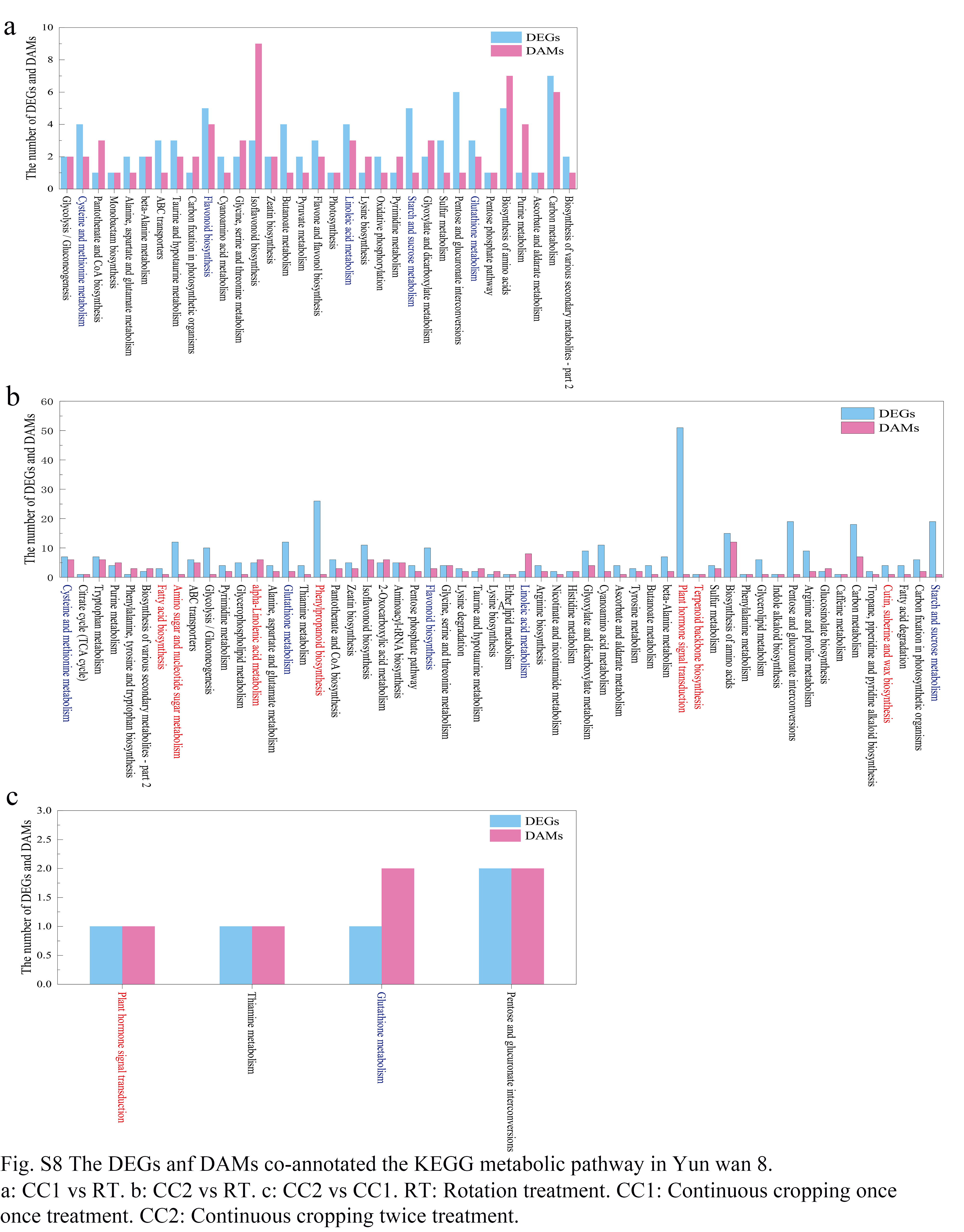

Supplement: Supplementary file 13 — Supplementary Material 13 [file 12870_2023_4225_MOESM13_ESM.tif]
